# Causal Multistate Models to Evaluate Treatment Delay

**DOI:** 10.1002/sim.70061

**Published:** 2025-04-08

**Authors:** Ilaria Prosepe, Saskia le Cessie, Hein Putter, Nan van Geloven

**Affiliations:** ^1^ Department of Biomedical Data Sciences Leiden University Medical Center Leiden the Netherlands; ^2^ Department of Clinical Epidemiology Leiden University Medical Center Leiden the Netherlands

**Keywords:** causal inference, g‐computation, multistate model, observational data, survival analysis

## Abstract

Multistate models allow for the study of scenarios where individuals experience different events over time. While effective for descriptive and predictive purposes, multistate models are not typically used for causal inference. We propose an estimator that combines a multistate model with g‐computation to estimate the causal effect of treatment delay strategies. In particular, we estimate the impact of strategies such as awaiting natural recovery for 3 months, on the marginal probability of recovery. We use an illness–death model, where illness and death represent, respectively, treatment and recovery. We formulate the causal assumptions needed for identification and the modeling assumptions needed to estimate the quantities of interest. In a simulation study, we present scenarios where the proposed method can make more efficient use of data compared to an alternative approach using cloning–censoring–reweighting. We then showcase the proposed methodology on real data by estimating the effect of treatment delay on a cohort of 1896 couples with unexplained subfertility who seek intrauterine insemination.

## 1 Introduction

Multistate models have gathered considerable attention in medical statistics, offering a pivotal extension of survival analysis methodology to settings where individuals may experience multiple different events over time [1, 2, 3]. Multistate models are primarily used for descriptive and predictive purposes, for example, describing the likelihood of a specific event occurring in the presence of intermediate or competing events or gaining insight into the association between prognostic factors and different transition probabilities. Transitions in the multistate model represent those observed in the data. Thus, when used on observational data, multistate models are not targeted toward answering causal questions that aim to estimate outcomes under “what if” scenarios resulting from potential interventions that change certain transitions in the model.

The value of estimating transition rates under hypothetical changes in event distributions was heavily debated in the survival analysis literature, as identification requires additional assumptions [4, 5, 6, 7, 8, 9]. In particular, survival in a hypothetical world where one competing cause of death is removed is identifiable under the assumptions that a realization of the latent failure times is unchanged by cause removal and that latent failure times are independent of one another [4, 5, 8]. Formulating these assumptions necessitates a comprehensive understanding of the mechanism under study and demands careful consideration of the setting in which the data were collected. This led many researchers to advocate for “sticking to this world” and methodological extensions of the multistate framework predominantly followed this advice by restricting to *observable* quantities rather than latent failure times [4, 5, 8, 10, 11].

It is nowadays increasingly recognized that when the research question is intrinsically causal, the analysis should reflect this, with careful description and assessment of the assumptions [12]. Some recent works applied a causal approach in a multistate setting. Gran et al. [13] investigated the effect of hypothetical interventions on return‐to‐work for a cohort of work rehabilitation participants, showing how multistate models can be employed to address causal questions: by including treatment/exposure as one of the states, by modeling treatment/exposure as a covariate that has an effect on some of the transitions or by reweighting the population to estimate the transition intensities in a different population. Valeri et al. [14] recently examined how inequities in access to healthcare contribute to racial disparities in the survival of cancer patients by employing a multistate model for the hypothetical scenario where a black person would have the same access to healthcare as a white person. Young et al. [15] formulated a causal framework for classical statistical estimands in failure time settings with competing events for contexts where treatment is assigned at baseline. Erdmann et al. [16] used multistate models to estimate treatment effects in the hypothetical scenario of a clinical trial where the treatment was never interrupted due to patients no longer being allowed to receive the investigational drug.

In this paper, we propose the use of an illness–death model, a specific type of multistate model, to estimate the causal effect of treatment delay from observational data in the presence of baseline confounders. In the proposed illness–death model, treatment and recovery represent, respectively, the analogue to illness and death. We provide estimands for when hypothetical modifications to the transition from the starting state to the treatment state are of interest. Investigating the impact of treatment delay has clear medical relevance, as it may help to prevent potentially expensive and invasive treatments for patients who stand a reasonable chance of recovering without intervention. However, the optimal delay of treatment initiation, often referred to as a “wait‐and‐see” or “expectant management” periods, remains largely unknown. Our multistate formulation shares similarities to that of Valeri et al. [14]. However, the estimands of interest and the formulation differ, as they approach their problem from a mediation analysis standpoint [14].

The rest of the paper is set up as follows: In Section 2, we provide a formal definition of the recovery probabilities in the hypothetical scenario of a fixed delay of treatment initiation, outline the assumptions required to identify this quantity from observational data in the presence of baseline confounding, and introduce our proposed estimation approach; in Section 3, we present a simulation study to assess the performance of our proposed method in small‐sample scenarios and compare it to the clone–censor–reweighting method [17, 18]; in Section 4, we apply our method to a cohort of couples with unexplained subfertility, studying the timing of intrauterine insemination (IUI). In Section 5, we discuss our findings.

## 2 Methods

### 2.1 Setting

**FIGURE 1:**
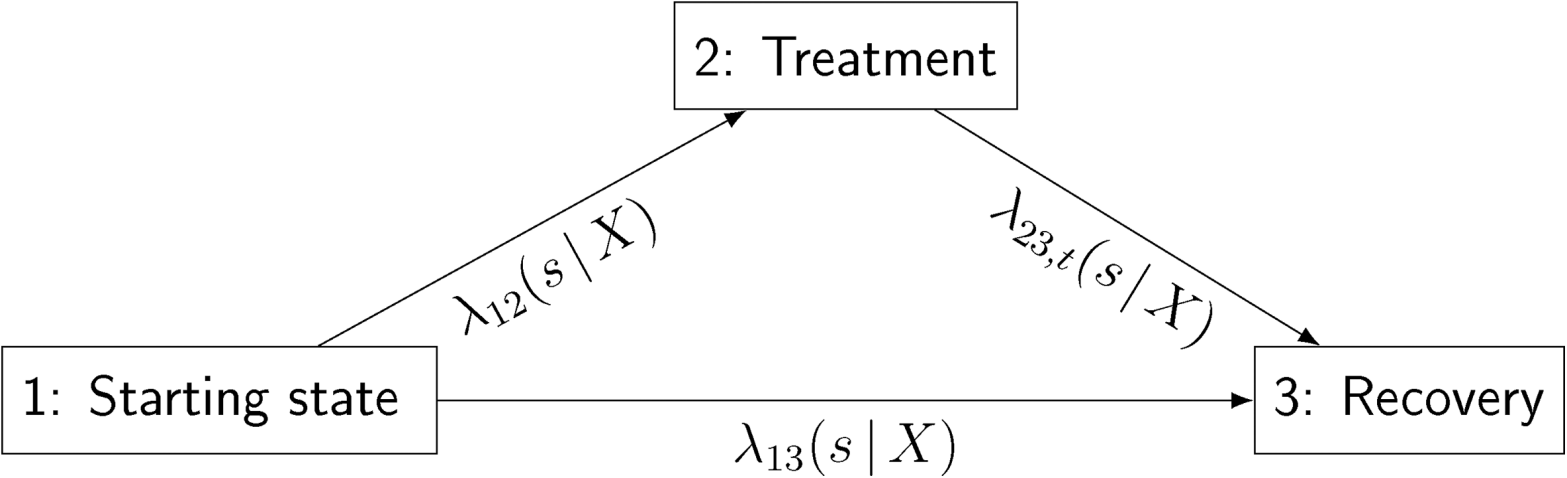
The illness–death setting: The three states (starting state, treatment, and recovery) are connected by arrows that represent the possible transitions between them. The respective transition intensities between states are depicted.

We consider the situation outlined in Figure 1. Initially, all patients are untreated at time 0 (starting state), which may correspond to diagnosis or, more generally, the moment when patients consult a doctor for guidance on whether and when to initiate treatment. At this point, doctor and patient choose their treatment strategy: some patients start treatment right away, while others choose to delay treatment initiation to first see if recovery without treatment occurs. In this context, patients may transition between three states: starting state (state 1), treatment (state 2), and recovery (state 3). Some move directly from the starting state to recovery, while others first transition from the starting state to treatment. Not all patients necessarily reach the recovery state: some may never leave the starting state, and some may never leave the treatment state. This setting is known as an irreversible *illness–death model* in multistate literature [2]. This paper focuses on studying treatment strategies of the form “if not recovered by a certain time, then initiate treatment.” We refer to the waiting time before treatment initiation as “treatment delay.”

### 2.2 Notation and Estimand

Let s denote the time since entry on the starting state (s=0). We let $\tilde{R}$ be the time from baseline to recovery. Some patients transition directly from the starting state to recovery, while others first receive treatment. We let $\tilde{W}$ be the time to first event (so either recovery if the patient recovers untreated or treatment if the patient receives treatment first). Following a *competing risks* notation, we define the indicator $\delta_{\tilde{W}}$ that takes value 1 if the patient is treated and takes value 2 if the patient recovers without treatment. We then let C be time from baseline to censoring. We let $R=\min\{\tilde{R},C\}$ be the observed recovery time, with status indicator $\delta_{R}$ that takes value 1 if we observe the recovery and 0 if the patient is censored before recovery. We let $W=\min\{\tilde{W},C\}$ be the observed first event with indicator $\delta_{W}$ that takes value 1 if the patient is treated, takes value 2 if the patient recovers without treatment, and takes value 0 if the patient is censored before either treatment or recovery. Times R and W depend on X, a vector of baseline covariates that confound the relation between treatment time and recovery.

We let g be a treatment strategy of the form “if not yet recovered by time $t_{g}$, then initiate treatment at $t_{g}$.” The strategy “never assign treatment” corresponds to $t_{g}=\infty$. We define $R^{g}$ as the potential recovery time under treatment strategy g. Our estimand of interest is $\text{Prob}(R^{g}<\ell )$, the marginal, that is, population averaged, recovery probability by a fixed time point ℓ under treatment strategy g.

### 2.3 Multistate Model

We define the (observable) hazard rates, also known as *transition intensities* [2], for the three transitions as

(1) $$\begin{array}{ll} \lambda_{12}(s|X) & =\lim_{\Delta s\to 0}\frac{\text{Prob}(s\leq W<s+\Delta s,\delta_{W}=1|W\geq s,X)}{\Delta s}\\ \lambda_{13}(s|X) & =\lim_{\Delta s\to 0}\frac{\text{Prob}(s\leq W<s+\Delta s,\delta_{W}=2|W\geq s,X)}{\Delta s}\\ \lambda_{23,t}(s|X) & =\lim_{\Delta s\to 0}\frac{\begin{array}{c} \text{Prob}\left(s\leq R<s+\Delta s,\delta_{R}=1|R\geq s,W=t,\delta_{W}=1,X\right) \end{array}}{\Delta s}\;s\geq t \end{array}$$

We define the cumulative hazard function for the i→j transitions as

(2) $$\begin{array}{lll} \Lambda_{1j}(s|X) & =\int_{0}^{s}\lambda_{1j}(u|X)du & j\in \{2,3\}\\ \Lambda_{23,t}(s|X) & =\int_{t}^{s}\lambda_{23,t}(u|X)du & s\geq t\; \end{array}$$

and the functions $S_{12}(s,X)$, $S_{13}(s,X)$ and $S_{23,r}(s\;|\;X)$ as

(3) $$\begin{array}{lll} S_{1j}(s|X) & =\exp(-\Lambda_{1j}(s|X)) & \\ & =\underset{u\leq s}{\pi }\left(1-\lambda_{1j}(u|X)\right)du & j\in \{2,3\}\\ S_{23,t}(s|X) & =\exp(-\Lambda_{23,t}(s|X)) & \\ & =\underset{t\leq u\leq s}{\pi }\left(1-\lambda_{23,t}(u|X)\right)du & s\geq t\; \end{array}$$

where $\pi_{t\leq u\leq s}$ indicates the product taken over the infinitesimally small intervals [s,s+Δs]. We remark that the functions $S_{1j}(s,X)$ (j∈{2,3}), while they can be estimated, cannot in general be interpreted as survival distributions. Only when the competing events (and the censoring process) are independent conditionally on X can these functions be interpreted as the survival distribution in the situation that the competing event does not occur. We refer to literature [2] for further background.

### 2.4 Identification of Causal Effects for Multistate Models

In the following, we demonstrate how our quantity of interest $\text{Prob}(R^{g}<\ell )$ is identifiable under four identifiability conditions: consistency and, conditional on a set of baseline covariates X, positivity, exchangeability, and independent censoring. These conditions, delineated in this section, represent an adapted version of the standard identifiability conditions used in causal inference for time‐to‐event outcomes [19], to match our setting of interest. Under these assumptions, the following identification formula holds:

(4) $$\begin{array}{ll} & \text{Prob}(R^{g}<\ell )=E_{X}\left\{\begin{array}{ll} 1-S_{13}(\ell |X) & \text{for }\ell \leq t_{g}\\ 1-S_{13}(t_{g}|X)\cdot S_{23,t_{g}}(\ell |X) & \text{for }\ell >t_{g} \end{array}\right. \end{array}$$

We will now formulate in detail the four identifiability conditions and prove Equation (4) under these conditions.

Let A(s) be the time‐dependent treatment indicator that takes the value 0 if the patient is still untreated at time s and 1 otherwise and let Ā(s) be the treatment history up to s: Ā(s)={A(u):u∈[0,s]}. Similarly, let C(s) be the time‐dependent censoring indicator that takes the value 0 if the patient is uncensored at time s and 1 otherwise, and let $\bar{C}(s)$ be the censoring history up to s: $\bar{C}(s)=\{C(u):u\in [0,s]\}$. Under this notation, the identifiability conditions are

1. **Consistency:** $R^{g}=(\tilde{R}\;|\;A(\tilde{R})=0)$ if $\tilde{R}\leq t_{g}$ and $R^{g}=(\tilde{R}\;|\;\tilde{W}=t_{g},\delta_{\tilde{W}}=1$) if $R>t_{g}$. This means that the counterfactual outcome $R^{g}$ is equal to the outcome $\tilde{R}$ on those subjects who actually follow the treatment strategy g.
2. **Positivity:** $\lim_{\Delta s\to 0}\frac{1}{\Delta s}\text{Prob}(t_{g}\leq W<t_{g}+\Delta s,\delta_{W}=1\;|\;W\geq t_{g},X)>0$.
  In other words, the treatment rate at time $t_{g}$ given X for subjects who have not yet recovered, have not yet been treated, and are not yet censored is larger than 0 for every treatment strategy g of interest and for all observed values of X. This also implies that there are still subjects at risk for transitions 1→2 and 1→3 at time $t_{g}$.
3. **Conditional Exchangeability:** for Δs→0,
$$\begin{array}{ll} & I(s\leq R^{g}<\Delta s)\;⊥⊥\;\bar{A}(s+\Delta s)|\tilde{R}\geq s,\\ & \;X,\{A(u)=I(u>t_{g})\forall u\in [0,s]\}. \end{array}$$
  In other words, within each level of X, the probability of being treated during the interval [s,s+Δs) is independent of the outcome under treatment strategy g in that same interval for all s≥0. The conditional exchangeability assumption requires that, within each level of the baseline confounder X, treated and untreated patients are comparable at each treatment time, meaning those who receive treatment are representative of those who do not (and vice versa). This assumption holds when the decision to initiate treatment is made based on baseline characteristics only, as outlined in Section 2.1. If the decision to initiate treatment is based on time‐varying confounders, this assumption is not met.
4. **Independent Censoring**: for Δs→0:
  - $I(s\leq \tilde{R}<\Delta s)\;⊥⊥\;\bar{C}(s+\Delta s)|\{A(u)=I(u>t_{g})\forall u\in [0,s]\},R\geq s,X$;
  - for $s\leq t_{g}$, $\bar{A}(s+\Delta s)\;⊥⊥\;\bar{C}(s+\Delta s)|W\geq s,X$.
  In other words, $\tilde{R}$ and the observed treatment strategy are independent of the censoring mechanism conditionally on X. Similarly to the conditional exchangeability assumption, the independent censoring assumption requires that, within each level of X, the patients who remain uncensored at each censoring time are representative of those who were censored.

Let us now denote by $\lambda^{g}(s\;|\;X)$ the counterfactual hazard rate of recovery under treatment strategy g:

(5) $$\lambda^{g}(s|X)=\underset{\Delta s\to 0}{\lim}\frac{\text{Prob}(s\leq R^{g}<s+\Delta s\;|\;R^{g}\geq s,X)}{\Delta s}$$

Under this notation

(6) $$\begin{array}{ll} \text{Prob}(R^{g}<\ell ) & =E_{X}\text{Prob}(R^{g}<\ell \;|\;X)\\ & =E_{X}\left(1-\underset{s\leq \ell }{\pi }\left(1-\lambda^{g}(s\;|\;X)ds\right)\right) \end{array}$$

where the first equality is due to the law of total expectation and the second follows by splitting the interval [0,s] into infinitesimally small sub‐intervals. By assuming consistency, positivity, and conditional exchangeability, for $s\leq t_{g}$, we have

(7) $$\begin{array}{rl} \lambda^{g}(s|X):= & \;\lim_{\Delta s\to 0}\frac{\text{Prob}(s\leq R^{g}<s+\Delta s|R^{g}\geq s,X)}{\Delta s}\\ \overset{\text{C.E.}}{=} & \;\lim_{\Delta s\to 0}\frac{\text{Prob}(s\leq R^{g}<s+\Delta s|R^{g}\geq s,A(s^{-})=0,X)}{\Delta s}\\ \overset{\text{Co}.}{=} & \;\lim_{\Delta s\to 0}\frac{\text{Prob}(s\leq R^{g}<s+\Delta s|\tilde{W}\geq s,X)}{\Delta s}\\ \overset{\text{C.E.}+\text{Co}.}{\;=} & \;\lim_{\Delta s\to 0}\frac{\text{Prob}(s\leq \tilde{W}<s+\Delta s,\delta_{\tilde{W}}=2|\tilde{W}\geq s,X)}{\Delta s}\\ \overset{\text{I.C}.}{=} & \;\lim_{\Delta s\to 0}\frac{\text{Prob}(s\leq \tilde{W}<s+\Delta s,\delta_{\tilde{W}}=2|W\geq s,X)}{\Delta s}\\ \overset{\text{I.C.+def.W}}{\;=} & \;\lim_{\Delta s\to 0}\frac{\text{Prob}(s\leq W<s+\Delta s,\delta_{W}=2|W\geq s,X)}{\Delta s}\;=:\;\lambda_{13}(s|X) \end{array}$$

Conditional exchangeability (C.E.) ensures the first equality as it allows us to condition on patients who are still untreated just before time s. Consistency (Co.) ensures the second equality as it allows us to switch from the condition $R^{g}\geq s$ to R≥s, which combined with A(s)=0 yields the condition W≥s. Conditional exchangeability ensures the third equality of Equation (7) as it allows restricting to patients who remain untreated during the next short amount of time Δs and, together with consistency, it ensures we can switch from the counterfactual $s\leq R^{g}<s+\Delta s$ to the factual $s\leq \tilde{W}<s+\Delta s,\delta_{\tilde{W}}=2$. Points (a) and (b) of the independent censoring (I.C.) assumption ensure that $\tilde{R}$, A(s) and C(s) are mutually independent and, therefore, that we can condition on patients who are still uncensored (and untreated) just before time s (fourth equality of Equation (7)). Points (a) and (b) of the independent censoring assumption (I.C.) ensure, together with $W=\min\{\tilde{W},C\}$, the fifth equality of Equation (7), as they allow restricting to patients who remain uncensored (and untreated) during the next short amount of time Δs. Positivity ensures that there are subjects at risk for transition 1→3 for all $s\leq t_{g}$, ensuring that $\lambda_{13}(s\;|\;X)$ exists for all $s\leq t_{g}$. This would not happen if, for example, patients with certain X values all received treatment before time $t_{g}$. Similarly, for $s>t_{g}$:

$$\begin{array}{rl} \lambda^{g}(s|X):= & \;\lim_{\Delta s\to 0}\frac{\text{Prob}(s\leq R^{g}<s+\Delta s|R^{g}\geq s,X)}{\Delta s}\\ \overset{\text{C.E.}}{=} & \;\lim_{\Delta s\to 0}\frac{\begin{array}{c} \text{Prob}(s\leq R^{g}<s+\Delta s|\tilde{W}=t_{g},\delta_{\tilde{W}}=1,R^{g}\geq s,X) \end{array}}{\Delta s}\\ \overset{\text{Co.}}{=} & \;\lim_{\Delta s\to 0}\frac{\begin{array}{c} \text{Prob}\left(s\leq \tilde{R}<s+\Delta s|\tilde{W}=t_{g},\delta_{\tilde{W}}=1,\tilde{R}\geq s,X\right) \end{array}}{\Delta s}\\ \;\overset{\text{I.C.+def.W}}{\;=} & \;\lim_{\Delta s\to 0}\frac{\begin{array}{c} \text{Prob}(s\leq R<s+\Delta s,\delta_{R}=1|W=t_{g},\delta_{W}=1,R\geq s,X) \end{array}}{\Delta s}\\ & =:\;\lambda_{23,t_{g}}(s|X) \end{array}$$

Conditional exchangeability ensures the first equality as it allows us to condition on patients who were treated at time $t_{g}$. Consistency ensures the second equality as it allows us to switch from the condition $R^{g}\geq s$ to R≥s. The independent censoring assumption ensures, together with $R=\min\{\tilde{R},C\}$, the equality of the quantities at lines 3 and 4. Positivity ensures that there are subjects that did transition from state 1 to state 2 at time $t_{g}$, ensuring that $\lambda_{23,t_{g}}(s\;|\;X)$ exists.

This means that when the identifiability conditions hold, the transition intensities $\lambda_{13}(s\;|\;X)$ and $\lambda_{23,t}(s\;|\;X)$ remain unchanged after the modification of transition 1→2. It follows that we can replace the quantity $\lambda^{g}(s\;|\;X)$ in Equation (6) by $\lambda_{13}(s\;|\;X)$ for $s\leq t_{g}$ and by $\lambda_{23,t_{g}}(s\;|\;X)$ for $s>t_{g}$, yielding Equation (4).

### 2.5 Estimation

We propose a semi‐parametric g‐computation, which relies on the correct specification of the outcome model, that is, of transition hazards $\lambda_{13}(s\;|\;X)$ and $\lambda_{23,t}(s\;|\;X)$. We model $\lambda_{13}(s\;|\;X)$ and $\lambda_{23,t}(s\;|\;X)$ using a Cox‐type model with a clock‐reset at the time of treatment initiation:

(8) $$\begin{array}{ll} \lambda_{13}(s\;|\;X) & =\lambda_{13,0}(s)\exp\{f_{13}(X;\beta_{13})\}\\ \lambda_{23,t}(s\;|\;X) & =\lambda_{23,t,0}(s-t)\exp\{f_{23}(X,t;\beta_{23},\gamma )\}\;\text{with}\;s\geq t \end{array}$$

where $\lambda_{13,0}(s)$ and $\lambda_{23,t,0}(s)$ represent baseline hazards for $\lambda_{13}(s\;|\;X)$ and $\lambda_{23,t}(s\;|\;X)$, respectively; $\beta_{13}$ is the (possibly time‐dependent) parameter vector for the effect of X on recovery without treatment; $\beta_{23}$ is the (possibly time‐dependent) parameter vector for the effect of X on recovery after treatment; γ is the parameter vector for the effect of treatment delay t on recovery after treatment; and $f_{13}(X;\beta_{13})$ and $f_{23}(X,t;\beta_{23},\gamma )$ are functions representing the (possibly time‐dependent) multiplication factor on the log scale for the hazards of transitions 1→3 and 2→3, respectively, for an individual with covariate values X. With the proposed semi‐parametric multistate method, we are less concerned about random violations of the positivity assumption. Unlike non‐parametric estimation of (4), this method allows us to borrow information across treatment strategies and levels of X, so it is not necessary to observe each treatment strategy g for the every level of X. Resetting the clock is helpful for treatment strategies where $t_{g}$ is close to 0. As all individuals start untreated, data may be insufficient for the correct estimation of $\lambda_{23,t_{g}}(s\;|\;X)$ with a clock‐forward approach. The flexibility in borrowing information comes at the expense of further assumptions, that is, the correct specification of $\lambda_{13}(s\;|\;X)$ and $\lambda_{23,t}(s\;|\;X)$. A simple choice for these hazards would be using Cox proportional hazards models: $\lambda_{13}(s\;|\;X)=\lambda_{13,0}(s)\exp\{\beta_{13}X\}$ and $\lambda_{23,t}(s\;|\;X)=\lambda_{23,t,0}(s-t)\exp\{\beta_{23}X+\gamma t\}$, where the parameter vectors $\beta_{13}$, $\beta_{23}$ and γ do not vary over time. This version of the hazards can be used if the assumptions of linearity and proportional hazards hold. Under this simple choice, the following estimator of (4) can be used:

(9) $$\begin{array}{ll} & \hat{\text{Prob}}(R^{g}<\ell )=\\ & E_{X}\left\{\begin{array}{ll} 1-\exp(-{\hat{\Lambda }}_{13,0}(\ell )\exp\{{\hat{\beta }}_{13}X\}) & \text{for }\ell \leq t_{g}\\ 1-\exp\left(-{\hat{\Lambda }}_{13,0}(t_{g})\exp\{{\hat{\beta }}_{13}X\}-\right.\left.{\hat{\Lambda }}_{23,t,0}(\ell -t_{g})\exp\{{\hat{\beta }}_{23}X+\hat{\gamma }t_{g}\}\right) & \text{for }\ell >t_{g} \end{array}\right. \end{array}$$

where the cumulative baseline hazards $\Lambda_{13,0}(s)=\int_{0}^{s}\lambda_{13,0}(u)du$ and $\Lambda_{23,t,0}(s-t)=\int_{t}^{s}\lambda_{23,t,0}(u)du$ can be estimated by means of the Breslow estimator and $E_{X}$ indicates that we average over the empirical distribution of X in the sample. Interactions can be included in the covariate set X if needed for correct model specification. If the linearity or the proportional hazard assumptions fail for a covariate, an alternative functional form of that covariate or an interaction of that covariate with an appropriate function of time could be employed. We refer to the literature [20] for further details on model selection.

## 3 Simulation

We report our simulation setup and results according to the ADEMP (Aims, Data generating mechanisms, Estimands, Methods, Performance measures) structure [21].

### 3.1 Simulation Setup

#### Aim

The aim of this simulation is to evaluate the small‐sample performance of our proposed method and, secondly, to compare accuracy and efficiency to clone–censor–reweighting, an existing method, based on inverse probability weighting, that could alternatively be used for the estimation of the effect of treatment delay from observational data [17, 18, 22].

#### Data Generating Mechanism

We generated four different scenarios. Scenario 1 is the base scenario. Each subsequent scenario differs from the base scenario by introducing one single modification in the data generating mechanism. In all presented scenarios, we generated the data in such a way that the assumptions of consistency, conditional exchangeability, and positivity, conditional on the covariate X, hold. Parameter choices are loosely based on the data application presented in Section 4. To allow for clearer interpretation of the results, each scenario presented here assumes constant baseline hazards, modeled with an exponential distribution. For comparison, results from the same four scenarios using time‐varying baseline hazards from a Weibull distribution are provided in Appendix A.

*Scenario 1—Base*: We generated data representing N=2500 patients. We generated one continuous baseline covariate X∼𝒩(0,1), which influences both time‐to‐treatment and time‐to‐recovery. We then generated:

- A latent time of recovery without treatment V with hazard 0.4·exp(−0.25X);
- A latent time of treatment T, drawn from a discrete distribution with P(T=0)=exp(−0.05·exp(0.25X)) and discrete hazard P(T=s|T≥s)=0.1·exp(0.25X) at times s={0.25,0.5,0.75,1}; if no treatment time T is drawn, we assume the patient remains untreated until the end of follow‐up;
- A latent post‐treatment time of recovery U with hazard 0.8·exp(−0.15X−0.25T), so that the hazard of recovery decreases if treatment is started later;
- A latent censoring time C with hazard 0.2·exp(0.1X).

The choice to generate discrete treatment times, and not continuous, for our base scenario was motivated by wanting to ensure practical positivity for all treatment strategies that we wished to study, which is especially needed for clone–censor–reweight approach. The observed final data set was made up of the following covariates: (i) the covariate X; (ii) time of first event W=min{T,V} with treatment status indicator $\delta_{T}=I(W=T)$; and (iii) time of recovery R=T+U if T<V and R=V if T≥V.

The remaining scenarios challenge different assumptions regarding the correct model specification. We modify the data generating mechanism for transition 2→3 to challenge our proposed multistate approach, which requires the correct specification of the outcome model, and we modify the transition 1→2 to challenge the clone–censor–reweight method, which requires the correct specification of the time‐to‐treatment model. The generating mechanism for transition 1→3 remains unchanged across all scenarios, as modifying transition 2→3 is enough to challenge models that require the correct specification of the outcome model.

*Scenario 2—Continuous Treatment Times*: Time of treatment T was drawn from a continuous distribution with exponential hazard function 0.4·exp(0.25X).

*Scenario 3—Non‐proportional Effect of* T *in Transition* 2→3: The effect of T on the log hazard for U was modeled by separate baseline hazards, one for each discrete treatment time T. The new hazard function of transition 2→3 followed a Weibull distribution, with the shape parameter that depends on T. This makes the proportional hazard assumption fail for transition 2→3 with respect to the variable T. We chose the shape parameter $\alpha_{T}=0.75+(0.5\cdot T)/1.5$, yielding the hazard $0.8\cdot \alpha_{T}{\left(s-T\right)}^{\alpha_{T}-1}\cdot \exp(-0.15X)$ at time s. This choice regarding the shape parameter $\alpha_{T}$ was made to obtain recovery rates that are similar to the base scenario, to make it easier to compare results across scenarios.

*Scenario 4—Non‐proportional Effect of* X *in Transition* 1→2: Instead of a constant effect ($\beta_{12}=0.25$) of X on the discrete hazard of transition 1→2, as in the base scenario, we used a time‐dependent effect $\beta_{12}(s)=6{\left(s-0.5\right)}^{2}-1$, which varies quadratically over time. This makes the proportional hazard assumption fail for transition 1→2 with respect to the covariate X.

#### Estimand

The estimand of interest was $\text{Prob}(R^{g}<\ell )$, for 0<ℓ≤1.5, with g a given treatment initiation strategy. In this simulation, we compared the following strategies: initiating treatment right away ($t_{g}=0$), at $t_{g}=0.25$, at $t_{g}=0.5$, at $t_{g}=0.75$ and not initiating treatment before time 1.5 (we will refer to this strategy as the “Never” strategy).

#### Methods

We compared the proposed multistate method to the clone–censor–reweight method [17, 18]. To gain deeper insight from this comparison, we considered two variants of each method: one variant assumes that treatment delay is a continuous variable and has a linear effect on the log hazard of the outcome model, while the other categorizes treatment delay and stratifies hazards by treatment delay. All methods rely on the causal identifiability conditions presented in Section 2.4.

*Multistate Continuous*: We fitted the multistate model using Cox proportional hazards models, with hazards $\lambda_{13}(s\;|\;X)$ and $\lambda_{23,t}(s\;|\;X)$ as specified in Equation (8). Treatment delay was included as a continuous variable in the model for the log hazard for the transition from treatment to recovery, assuming linearity. Recovery probabilities under different treatment strategies were then estimated as presented in Equation (9). For this method, we need correct specification of $\lambda_{13}(s\;|\;X)$ and $\lambda_{23,t}(s\;|\;X)$.

*Multistate Categorical*: We modeled $\lambda_{23,t}(s\;|\;X)=\lambda_{23,t,0}(s-t)\exp\{\beta_{23}X\}$, stratifying the baseline hazard $\lambda_{23,t,0}(s-t)$ by treatment delay t. In scenario 2 where time to treatment is continuous, treatment delay t was categorized into $t_{\text{cat}}$, with levels [0,0.125], [0.125,0.375], [0.375,0.625], [0.625,0.875], [0.875,1.125], [1.125,1.5]. Patients who received treatment after 1.5 (time horizon) were censored at the time of treatment to avoid creating baseline hazards based on too few or too incomparable individuals (which are unnecessary for estimating our target estimand). The choice of intervals was aimed at obtaining similar results to scenario 1. This method also relies on the correct specification of $\lambda_{13}(s\;|\;X)$ and $\lambda_{23,t}(s\;|\;X)$.

*Clone–Censor–Reweight Categorical*: We performed the following: (i) cloning: each individual was assigned to one or more treatment strategies at time zero by creating clones, one for each treatment strategy that was compatible with their observed data at time zero; (ii) censoring: clones were artificially censored when they deviated from their assigned strategy; and (iii) reweighting: inverse probability weighting was used to address the dependent censoring we introduced in the data. For the estimation of the weights, a time‐to‐treatment Cox model was used, assuming a linear and proportional effect of X. In the simulation runs where infinite weights were produced, weights were trimmed to the 97.5 percentile. After cloning, censoring, and reweighting, we estimated the recovery probabilities by means of a (reweighted) Kaplan–Meier estimator. In scenario 2, where time to treatment was continuous, we introduced grace windows spanning 0.125 before and after the target treatment delay, effectively changing the estimand slightly. For example, treatment strategy “start at 0” became “start before time 0.125” and treatment strategy “start at 0.5” became “start between 0.375 and 0.625.” This method relies on the correct specification of the time‐to‐treatment model.

*Clone–Censor–Reweight Continuous*: After cloning, censoring, and reweighting the observational data as described in the previous method, the recovery probabilities were estimated via a (reweighted) Cox model. In the Cox model, the baseline hazard was stratified by treatment yes/no and treatment delay was included linearly as a single continuous variable for the stratum “treatment = yes.” Time was reset after treatment initiation. For scenario 2, grace windows spanning 0.125 before and after the target treatment delay were used for cloning, censoring, and reweighting. In the estimation of the reweighted Cox model, the treatment delay variable took the value associated with the treatment strategy of each clone (e.g., for clones who are consistent with the “start before 0.125” grace period, we used treatment delay equal to 0; for clones consistent with “start between 0.375 and 0.625” strategy, we used treatment delay equal to 0.5). Unlike the categorical method, this approach did not change the estimand, as we used the Cox model to estimate the probability of recovery for specific point values of treatment delay. This approach relies on the correct specification of both the time‐to‐treatment model and the Cox model and assumes that individuals who start treatment within the grace window can be combined together into a single group.

#### Performance Measures

Performance of the four methods was assessed numerically through bias and root‐mean‐square error (RMSE) at time horizon (1.5) and visually by comparing the true and estimated recovery probabilities along a fine grid of time horizons between 0 and 1.5. The truth was computed as the numerical integral $\int_{-\infty }^{\infty }\text{Prob}(R^{g}<\ell \;|\;X=x)f_{X}(x)dx$, where $f_{X}(x)$ is the underlying probability density function of X used in the data generation. We used the hazard $0.8\cdot \exp(-0.15X-0.25t_{g})$ as the true latent post‐treatment hazard of recovery under treatment strategy g.

### 3.2 Software

All analyses were conducted using the statistical software R (version 4.3.1) [23] using the packages mstate [24], survival [25], tidyverse [26], and matrixStats [27]. Our simulation code is available at https://github.com/survival‐lumc/CausalMultistate.

### 3.3 Simulation Results

**FIGURE 2:**
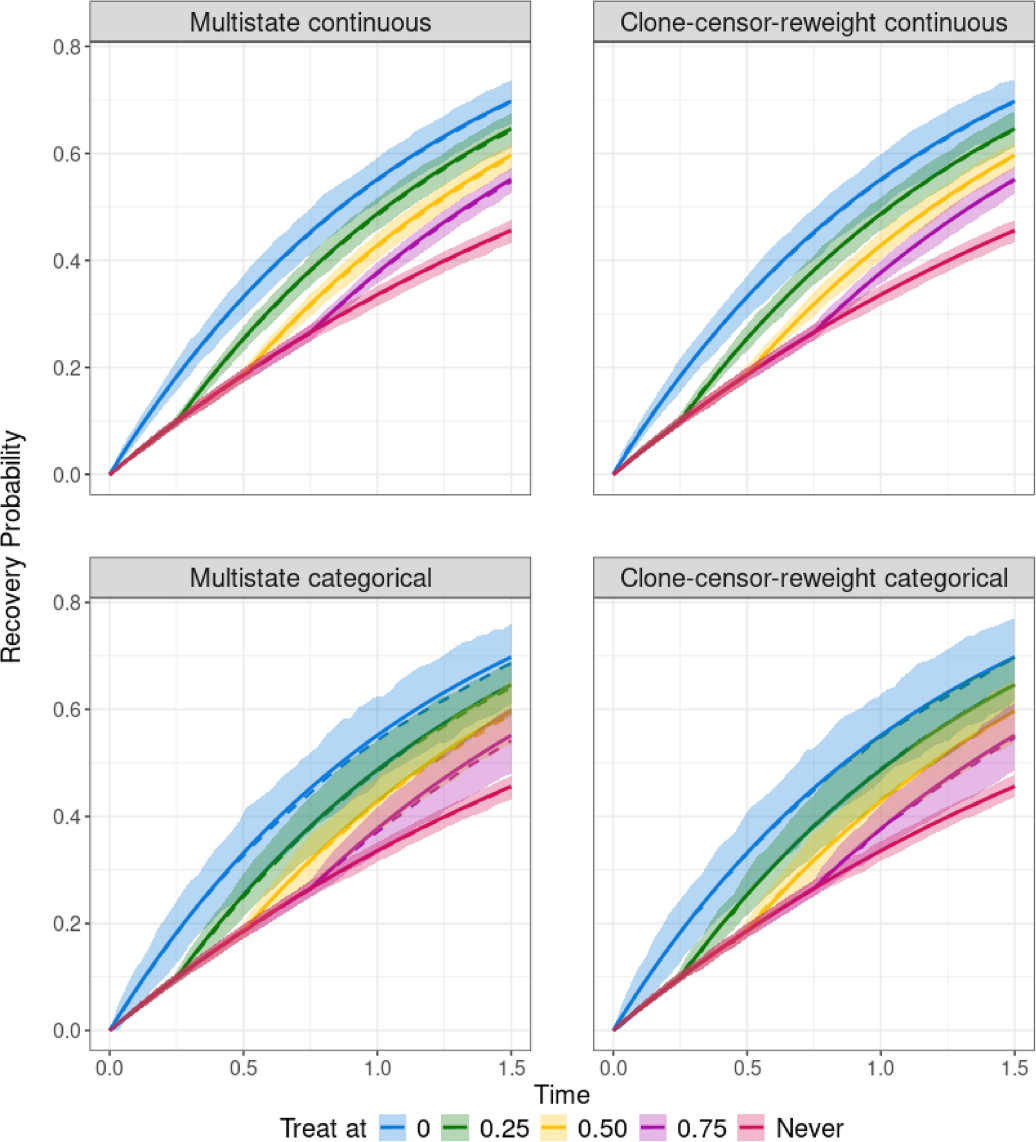
Visual representation of the simulation results for scenario 1, where assumptions needed for all estimation approaches are met. Cumulative recovery probabilities until time 1.5 are plotted under five treatment strategies: initiate immediately, delayed at 0.25, delayed at 0.5, delayed at 0.75, and never initiate. Four estimation approaches are reported: (i) *Multistate continuous*; (ii) *Multistate categorical*; (iii) *Clone–censor–reweight continuous*; (iv) *Clone–censor–reweight categorical*. The solid lines represent the true values, the dotted line represents the mean of the estimates, and the shaded area represents the [5th–95th] percentile of the estimates.

We ran our simulation 200 times. Table 1 provides numerical assessment of bias and RMSE at time horizon 1.5 for all four scenarios. Figure 2 provides a visual representation of the simulation results for the base scenario (scenario 1). Figures for the other scenarios can be found in Appendix A.

**TABLE 1:** Simulation results.

		Multistate continuous		Multistate categorical		CCR continuous		CCR categorical	
Scenario	Strategy	Bias	RMSE	Bias	RMSE	Bias	RMSE	Bias	RMSE
1‐Base: Correctly	0 months	0.00	0.03	−0.01	0.05	0.00	0.03	0.00	0.05
specified models	3 months	0.00	0.02	−0.01	0.03	0.00	0.02	0.00	0.03
	6 months	0.00	0.02	−0.01	0.04	0.00	0.02	0.00	0.03
	9 months	0.00	0.01	−0.01	0.04	0.00	0.02	0.00	0.04
	1 year	0.00	0.02	0.00	0.03	0.00	0.02	0.00	0.03
	Never	0.00	0.01	0.00	0.01	0.00	0.01	0.00	0.01
2‐Continuous	0 months	0.00	0.02	−0.01	0.05	−0.01	0.03	−0.02	0.05
treatment times	3 months	0.00	0.02	0.00	0.03	0.00	0.03	−0.01	0.04
	6 months	0.00	0.02	0.00	0.04	0.00	0.02	−0.01	0.04
	9 months	0.00	0.01	0.00	0.03	0.00	0.02	−0.01	0.04
	1 year	0.00	0.01	0.00	0.03	0.00	0.02	−0.01	0.03
	Never	0.00	0.01	0.00	0.01	0.00	0.01	0.00	0.01
3‐Non‐proportional	0 months	0.01	0.02	−0.01	0.04	−0.01	0.03	0.00	0.05
effect of T *in*	3 months	0.00	0.02	−0.01	0.03	−0.01	0.02	0.00	0.03
transition 2→3	6 months	0.00	0.02	0.00	0.04	0.00	0.02	0.00	0.04
	9 months	0.01	0.02	0.00	0.03	0.01	0.02	0.00	0.03
	1 year	0.02	0.03	0.00	0.03	0.04	0.04	0.00	0.03
	Never	0.00	0.01	0.00	0.01	0.00	0.01	0.00	0.01
4‐Non‐proportional	0 months	0.00	0.02	−0.01	0.05	0.03	0.04	−0.01	0.05
effect of X in	3 months	0.00	0.02	0.00	0.03	0.01	0.02	0.03	0.04
transition 1→2	6 months	0.00	0.01	0.00	0.03	0.00	0.02	0.02	0.04
	9 months	0.00	0.01	0.00	0.03	−0.02	0.02	−0.01	0.04
	1 year	0.00	0.01	0.00	0.02	−0.02	0.03	−0.03	0.04
	Never	0.00	0.01	0.00	0.01	−0.01	0.01	−0.01	0.01

*Note*: Bias and root‐mean‐squared error (RMSE) averaged over 200 simulation runs for the estimates of the cumulative recovery probabilities at time 1.5 under six treatment strategies: initiate immediately, delayed at 0.25, delayed at 0.5, delayed at 0.75, delayed at 1, and never initiate (before 1.5). Four estimation approaches are reported: (i) *Multistate continuous*; (ii) *Multistate categorical*; (iii) *Clone–censor–reweight (CCR) continuous*; (iv) *CCR categorical*. Four scenarios are presented: (1) assumptions needed for all estimation approaches are met; (2) time of treatment is generated continuously; (3) the effect of treatment delay on time‐to‐recovery is non‐proportional; and (4) the effect of the covariate X on time‐to‐treatment is non‐proportional. Standard errors (SEs) of the simulation can be derived using the relation RMSE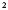 = SE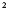 + Bias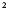.

In the base scenario (scenario 1), all methods performed well. This was expected, as data were generated in a way such that assumptions needed for all four estimation approaches were met. The *multistate continuous* model showed the smallest [5th–95th] percentile range of the estimates in Figure 2 and smallest RMSEs in Table 1. The *clone–censor–reweight continuous* ranked second in terms of RMSE.

In scenario 2, where treatment initiation was generated continuously, the *multistate continuous* yielded the smallest bias and RMSE, while the *clone–censor–reweight categorical* method presented bias for strategies where treatment was initiated (see Table 1 and Figure 4 in Appendix A). This is consistent with the fact that this method targeted a slightly different estimand. In 47 out of the 200 scenarios examined, the *clone–censor–reweight* method produced infinite weights, which were trimmed. This observation aligns with clone–censor–reweighting being more susceptible to random violations to positivity, which is common when treatment initiation is a continuous variable.

In scenario 3, where treatment delay influenced the log hazard of the recovery chances non‐proportionally, the *multistate continuous* and the *clone–censor–reweight continuous* showed bias due to incorrect model specification (see Table 1 and Figure 5 in Appendix A). For these methods, the RMSEs at 1.5 increased compared to scenario 1. For the other two methods, the RMSEs remained unchanged from scenario 1.

In the fourth scenario, where the covariate X influenced the log hazard of the recovery chances non‐proportionally, the two *clone–censor–reweight* approaches showed increased bias and RMSE compared to the base scenario as the model for the weights was not correctly specified.

## 4 Data Application

We applied the proposed method to estimate the probability of getting pregnant within 1.5 years after workup completion under different treatment delay strategies for patients with unexplained subfertility, defined as having tried to conceive naturally for over a year without success [28, 29] despite having parameters of infertility within normal ranges. The interest in determining whether and for how long to delay treatment initiation stems from the fact that while treatment usually increases the pregnancy probabilities [30, 31, 32], it also carries potential negative side effects associated with IUI, such as the potential risks of the hormonal therapy accompanying IUI and the financial and psychological burden on the couple.

We used data from a prospective cohort that was recruited across 38 hospitals in The Netherlands between January 2000 and October 2005. A more detailed description of the protocol and of the clinical definitions and setting can be found elsewhere [33, 34]. For the current study, we included couples with unexplained subfertility from seven (out of 38) centers that additionally collected data on IUI. These centers included 1896 couples, with at most 4 years of follow‐up. Of these couples, 569 became pregnant without treatment and 863 received IUI treatment. Cumulative probabilities over the time of pregnancy without treatment and of IUI treatment can be found in Appendix B. Of the 863 couples who received treatment, 163 became pregnant after treatment.

We set time 0 at the completion of workup, marking the start of “expectant management” (starting state, state 1). Some couples successfully achieved pregnancy (recovery, state 3) within 1.5 years, whereas others remained in either state 1 (they did not start treatment and did not conceive within 1.5 years) or 2 (they started treatment but did not conceive within 1.5 years). Treatment initiation could occur at any time during follow‐up, making it a continuous variable.

The estimands of interest were the probability of getting pregnant by 1.5 years if treatment was (i) initiated at 0 months; (ii) initiated at 6 months; and (iii) not initiated within 1.5 years. We included in the model the baseline covariates that were tested during the couples' workup and which may influence the future pregnancy probability as well as the choice to start IUI quickly or delay its initiation. These covariates are female age, subfertility duration, gynecologist referral (yes/no), infertility type (primary = no pregnancies before/secondary = lasting pregnancy before), fallopian tubal blockage (no blockage, one‐sided blockage, no test), and percentage of progressive sperm count. The covariate age and progressive sperm count were centered and standardized for the analysis.

We modeled the two outcome transitions 1→3 and 2→3 by means of a Cox proportional hazards model, assuming proportional hazards and linearity. Confidence intervals were obtained by bootstrapping.

We relied on the assumptions of consistency, positivity, conditional exchangeability, conditional independent censoring, and correct model specification. While the assumptions of consistency and conditional exchangeability are not testable, they were plausible in our context. The well‐defined delay periods supported the assumption of consistency, and the fact that no further evaluations were performed on these couples after workup completion supported the assumption of conditional exchangeability. We evaluated the validity of the positivity assumption in the data. Details can be found in Appendix B. The assumption of a conditionally independent censoring mechanism, although it could not be checked in the data, also appeared plausible. In our analysis, couples were mainly censored to start in vitro fertilization (IVF). The decision to start with IVF was largely influenced by the covariates in the dataset, and thus conditional independence seemed plausible to assume [35]. For the correct model specification assumption in the multistate model, we checked the assumptions of linearity (by plotting Martingale residuals) and proportional hazards (by testing the Schoenfeld residuals) for both transitions 1→3 and 2→3. Based on the linearity checks, subfertility duration and treatment delay were log‐transformed before being used as covariates. No notable violations of the proportional hazards assumption were found. We assessed the completeness of follow‐up for the study population over 1.5 years for both transitions 1→3 and 2→3 by means of a reverse Kaplan–Meier to ensure that we had an adequate number of individuals in the at‐risk set to at all time points. Figures and further details can be found in Appendix B.

**FIGURE 3:**
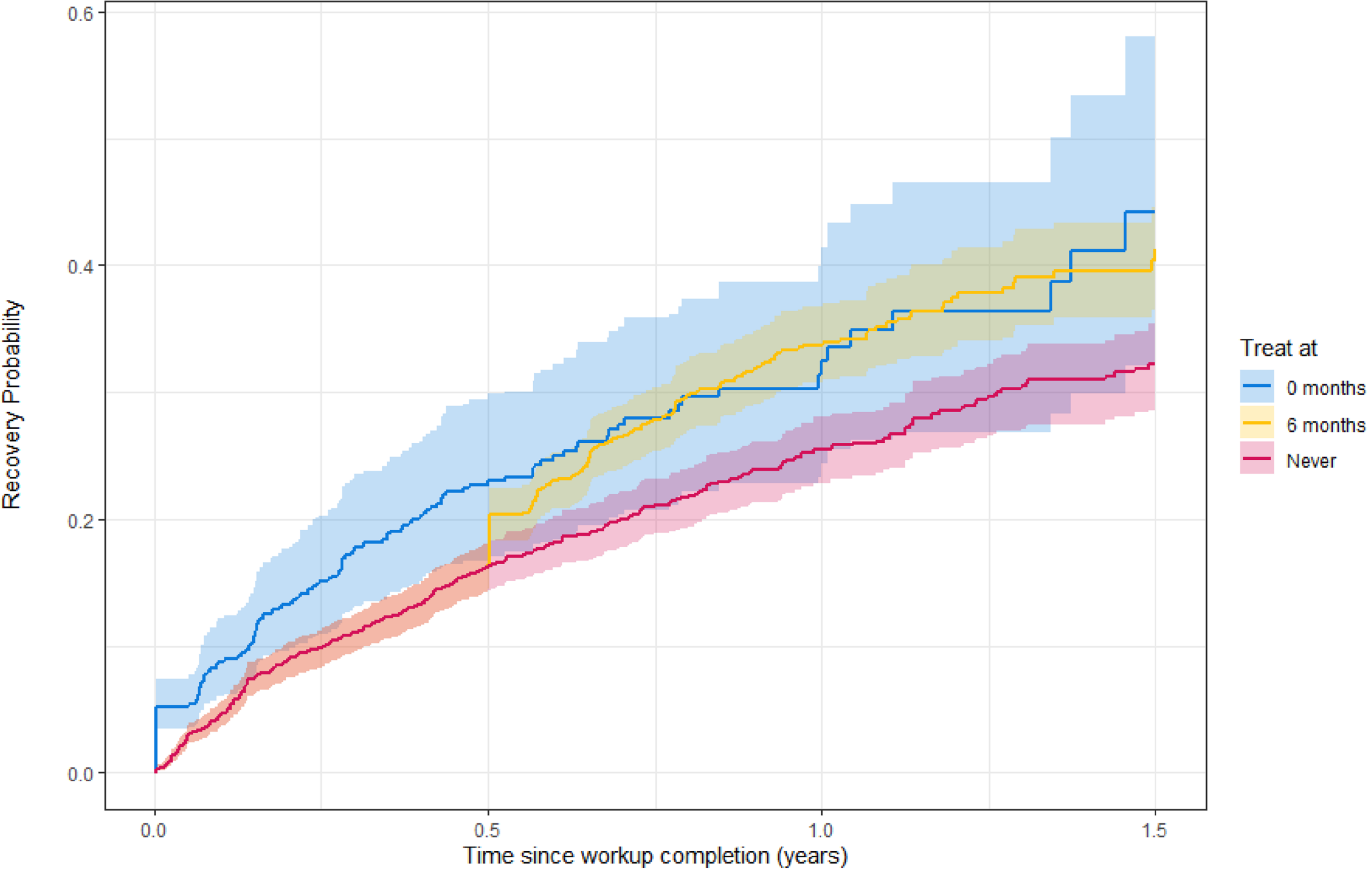
Estimated cumulative pregnancy probability for unexplained subfertile couples if they are assigned different treatment strategies.

Figure 3 shows the results of our analysis. Our findings suggest that, on average, initiating treatment directly or delaying treatment for 6 months yields similar pregnancy probabilities at 1.5 years post‐workup. This implies that delaying treatment does not significantly diminish the likelihood of pregnancy at 1.5 years while reducing the number of couples undergoing treatment. Not initiating IUI treatment before 1.5 years leads to lower pregnancy probabilities at 1.5 years. However, the wide confidence intervals prevent us from firm conclusions. For the “treat at 0 months” strategy, confidence intervals are particularly wide: this aligns with transition 2→3 having only a few subjects that were followed up for 1.5 years.

## 5 Discussion

In this paper, we propose an illness–death model combined with g‐computation that allows estimating the impact of treatment delay in observational data where all patients commence without treatment, and the intended treatment delay remains unobserved for patients who recover before starting treatment. Illness–death modeling provides a natural framework for this semi‐competing risk problem. The strength of our work lies in the careful formulation of the set of assumptions under which it is possible to use an illness–death model to draw causal conclusions on the effect of treatment delay. As a key finding, we demonstrated that the identifiability conditions commonly used in causal inference—consistency, positivity, and conditional exchangeability—imply, in the presented illness–death model, that other transition rates remain unchanged after modifying the transition to treatment. While survival analysis experts, as noted in the introduction, often caution against assuming that other transition rates are unaffected by such modifications, we formally show that, under these identifiability conditions, the transition rates for 1→3 and 2→3 indeed remain unchanged when the transition 1→2 is modified, as detailed in Section 2.4. The identifiability conditions provide a more intuitive framework compared to directly assuming that transition rates remain unchanged.

In our proposed estimation approach, we reset the clock after treatment and include treatment delay as a covariate. When using illness–death models, researchers can generally choose between a *clock‐forward* or *clock‐reset* approach, corresponding to Markov and Markov renewal models, respectively, and decide whether to include the time of arrival in the state as a covariate, which further relaxes the Markov (renewal) assumption. As remarked in the paper by Putter et al. [2], these modeling choices should primarily be informed by the clinical context. Our modeling choice was motivated by two main considerations. First, in our data application, the more relevant time scale after treatment is time since treatment. Second, data may be insufficient for a correct estimation of the hazard of transition 2→3 with a clock‐forward approach, as noted in Section 2.5. It is important to highlight that transition 2→3 is the only transition for which these modeling choices require careful evaluation. The other two transitions, which create a competing risk scenario, are always Markovian due to the absence of prior event history [2].

With simulations, we compared the performance of our proposed method to the clone–censor–reweight method. Both approaches are expected to provide asymptotically unbiased estimates of the probability of recovery under different treatment strategies when their respective assumptions are met. We evaluated scenarios in which the modeling assumptions for both methods held, as well as cases where these assumptions were violated, illustrating where and how each method may fail in limited sample sizes. Our proposed method may fail when either one (or both) of the transitions to the outcome is misspecified, whereas the clone–censor–reweight method may fail when the time‐to‐treatment model for the weights is misspecified. In the scenarios where the modeling assumptions for both methods hold, our multistate model exhibits greater efficiency (smaller variance) in utilizing data compared to the clone–censor–reweight approach, as it borrows information across different delay strategies for both the effect of the covariates and the effect of treatment delay. This finding is consistent with the existing literature, where g‐computation typically outperforms inverse probability weighting methods in terms of efficiency [36, 37, 38]. Double robust methods are able to provide consistent estimates when either the model for the weights or the outcome model is correctly specified. Exploring double‐robust methods for estimating treatment delay could be a promising avenue for future research.

A limitation of our method is that it does not account for time‐varying confounding, which occurs in situations where treatment decisions are influenced by prognostic factors beyond baseline characteristics. This happens, for instance, if patients are monitored regularly and treatment decisions are made based on their current health status. Our data application provided an ideal setting to demonstrate our methodology, as treatment initiation decisions were made using only baseline information. Exploring extensions of the current methodology to incorporate time‐varying confounders could be a potentially valuable direction for future research.

A second limitation is that our method does not account for competing events, which are relevant when patients can reach a state where treatment is no longer an option. For example, if treatment time was initially planned but the patient later developed conditions preventing them from receiving it, the current approach would need adaptation. In our data application, no such events occurred. While death and treatment ineligibility could in principle be competing events, their probability within our target population (couples trying to conceive with unexplained subfertility) was negligible, with no occurrences in our dataset. A possible extension of our approach could involve defining a new treatment strategy of interest, such as “treat at $t_{g}$ if not yet recovered and no competing event has occurred”, with the cumulative incidence of recovery if everyone followed treatment strategy g as the estimand. Extending identifiability conditions to this scenario warrants further exploration.

## Conflicts of Interest

The authors declare no conflicts of interest.

## Supporting information




**Data S1.** Supplement Materials.

**Data S1.** Data Files.
