# Supplementary material for: Causal Multistate Models to Evaluate Treatment Delay
Source: Stat Med. 2025 Apr 8;44(7):e70061. doi: 10.1002/sim.70061 (PMC11978571; doi:10.1002/sim.70061)
Supplement: Supplementary file 1 — Data S1. Supplement Materials. [file SIM-44-0-s001.pdf]

A Simulation study

A.1 Scenarios 2-4: Exponential distribution

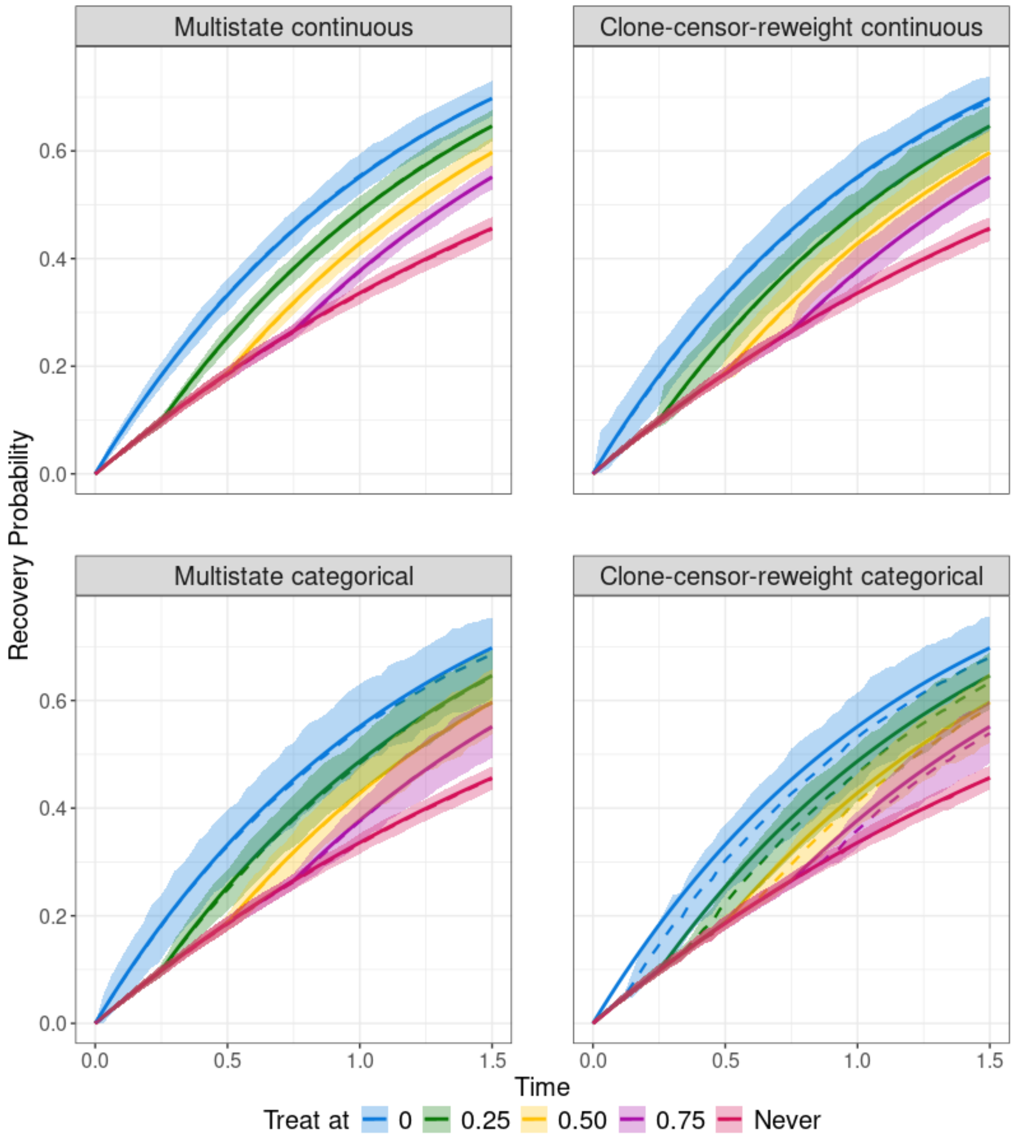

Figure 4: Visual representation of the simulation results for scenario 2. Cumulative recovery probabilities until time 1.5 are plotted, under five treatment strategies: initiate immediately, delayed at 0.25, delayed at 0.5, delayed at 0.75, and never initiate. Four estimation approaches are reported: (i) *Multistate continuous*; (ii) *Multistate categorical*; (iii) *Clone-censor-reweight continuous*; (iv) *Clone-censor-reweight categorical.* The solid lines represent the true values, the dotted line represents the mean of the estimates and the shaded area represents the $[5th - 95th]$ percentile of the estimates.

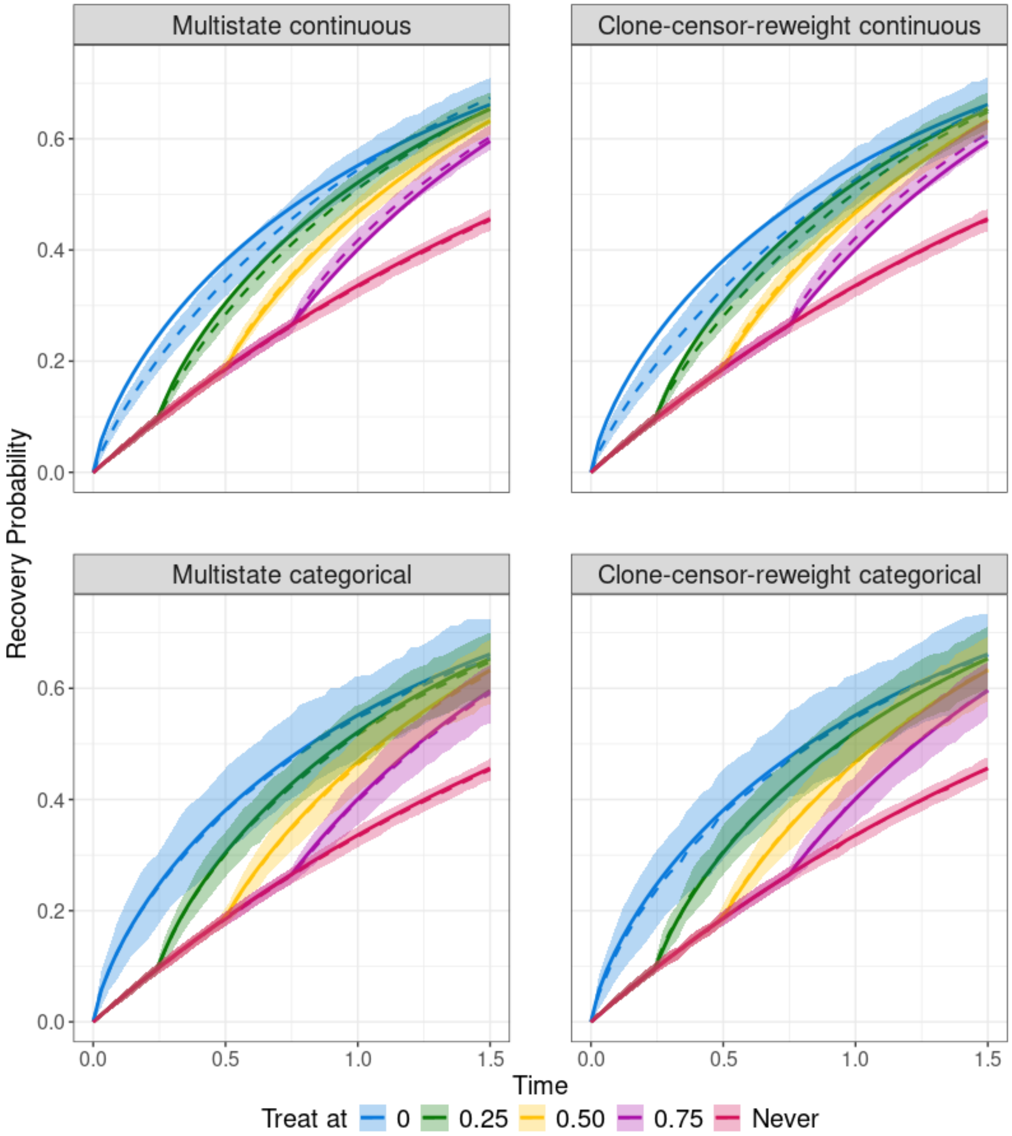

Figure 5: Visual representation of the simulation results for scenario 3. Cumulative recovery probabilities until time 1.5 are plotted, under five treatment strategies: initiate immediately, delayed at 0.25, delayed at 0.5, delayed at 0.75, and never initiate. Four estimation approaches are reported: (i) *Multistate continuous*; (ii) *Multistate categorical*; (iii) *Clone-censor-reweight continuous*; (iv) *Clone-censor-reweight categorical*.. The solid lines represent the true values, the dotted line represents the mean of the estimates and the shaded area represents the $[5th - 95th]$ percentile of the estimates.

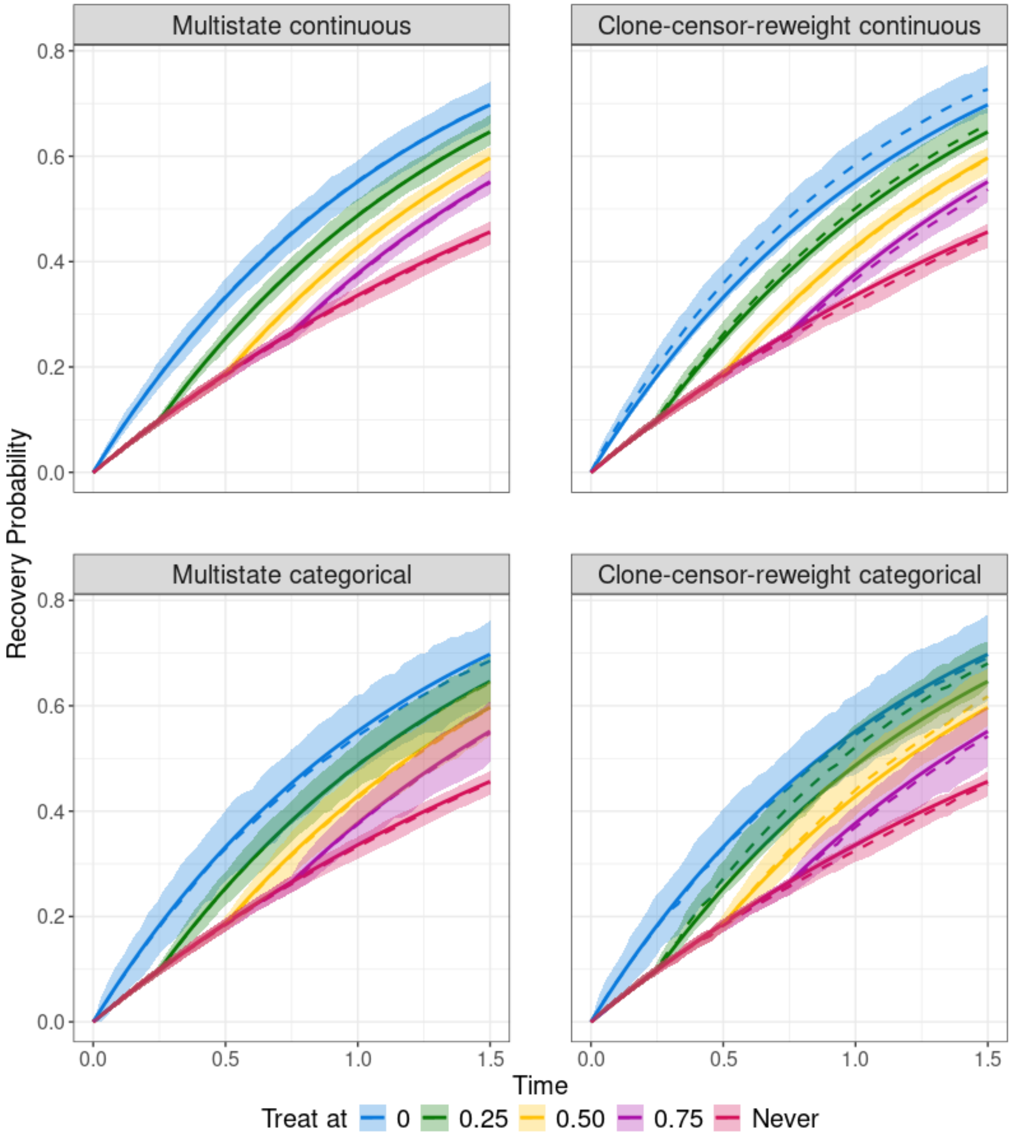

Figure 6: Visual representation of the simulation results for scenario 4. Cumulative recovery probabilities until time 1.5 are plotted, under five treatment strategies: initiate immediately, delayed at 0.25, delayed at 0.5, delayed at 0.75, and never initiate. Four estimation approaches are reported: (i) *Multistate continuous*; (ii) *Multistate categorical*; (iii) *Clone-censor-reweight continuous*; (iv) *Clone-censor-reweight categorical.* The solid lines represent the true values, the dotted line represents the mean of the estimates and the shaded area represents the $[5th - 95th]$ percentile of the estimates.

A.2 Weibull distribution

Data generating mechanism:
Similarly to the exponential case of the simulation study, we generated four different scenarios. Scenario 1 is the base scenario. Each subsequent scenario differs from the base scenario by introducing one single modification in the data generating mechanism. In all presented scenarios, we generated the data in such a way that the assumptions of consistency, conditional exchangeability and positivity, conditional on the covariate X, hold.

Scenario 1 - Base: We generated data representing $N = 2500$ patients. We generated one continuous baseline covariate $X \sim \mathcal{N}(0, 1)$, which influences both time-to-treatment and time-to-recovery.We then generated:

- a latent time of recovery without treatment V with hazard $0.4 \cdot s^{0.8} \cdot \exp(-0.25X)$ at time s;
- a latent time of treatment T, drawn from a discrete distribution with $P(T = 0) = \exp(-0.4 \cdot 0.125^{0.8} \cdot \exp(0.25X))$ and discrete hazard $P(T = s \,|\, T \geq s) = 0.4 \cdot \exp(0.25X) \cdot ((s + 0.125)^{0.8} - (s - 0.125)^{0.8}))$ at times $s = \{0.25, 0.5, 0.75, 1\}$; if no treatment time T is drawn, we assume the patient remains untreated until the end of follow-up;
- a latent post-treatment time of recovery U with hazard $0.8 \cdot s^{0.8} \cdot \exp(-0.15X - 0.25T)$ at time s, so that the hazard of recovery decreases if treatment is started later;
- a latent censoring time C with hazard $0.2 \cdot s^{0.8} \cdot \exp(0.1X)$ at time s.

Scenario 2 - Continuous treatment times: Time of treatment T was drawn from a continuous distribution with hazard function $0.4 \cdot s^{0.8} \cdot \exp(0.25X)$ at time s.

Scenario 3 - Non-proportional effect of T in transition $2 \to 3$: The effect of T on the log hazard for U was modelled by separate baseline hazards, one for each discrete treatment time T. The new hazard function of transition $2 \to 3$ followed a Weibull distribution, with the shape parameter that depends on T. This makes the proportional hazard assumption fail for transition $2 \to 3$ with respect to the variable T. We chose the shape parameter $\alpha_T = 0.75 + (0.5 \cdot T)/1.5$, yielding the hazard $0.8 \cdot \alpha_T (s - T)^{\alpha_T - 1} \cdot \exp(-0.15X)$ at time s. This choice regarding the shape parameter α_T was made to obtain recovery rates that are similar to the base scenario, to make it easier to compare results across scenarios.

Scenario 4 - Non-proportional effect of X in transition $1 \to 2$: Instead of a constant effect ($\beta_{12} = 0.25$) of X on the discrete hazard of transition $1 \to 2$, as in the base scenario, we used a time-dependent effect $\beta_{12}(s) = 6(s - 0.5)^2 - 1$, which varies quadratically over time. This makes the proportional hazard assumption fail for transition $1 \to 2$ with respect to the covariate X.

Results:
As shown in Figures 6–10, the same considerations discussed in the main text for the exponential distribution apply equally to the case with a Weibull baseline distribution.

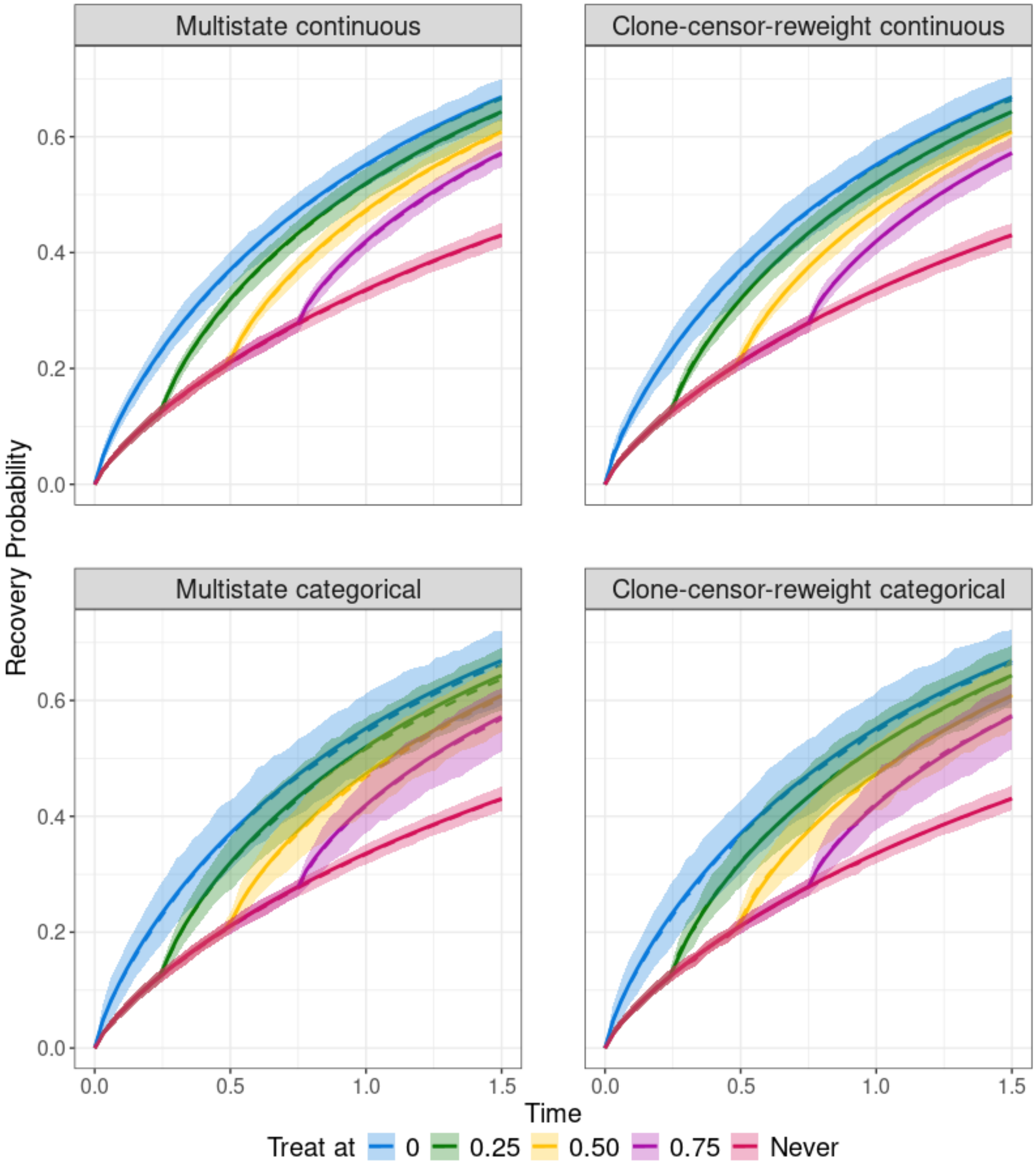

Figure 7: Visual representation of the simulation results for scenario 1, where assumptions needed for all estimation approaches are met. Cumulative recovery probabilities until time 1.5 are plotted, under five treatment strategies: initiate immediately, delayed at 0.25, delayed at 0.5, delayed at 0.75, and never initiate. Four estimation approaches are reported: (i) *Multistate continuous*; (ii) *Multistate categorical*; (iii) *Clone-censor-reweight continuous*; (iv) *Clone-censor-reweight categorical*. The solid lines represent the true values, the dotted line represents the mean of the estimates and the shaded area represents the $[5th - 95th]$ percentile of the estimates.

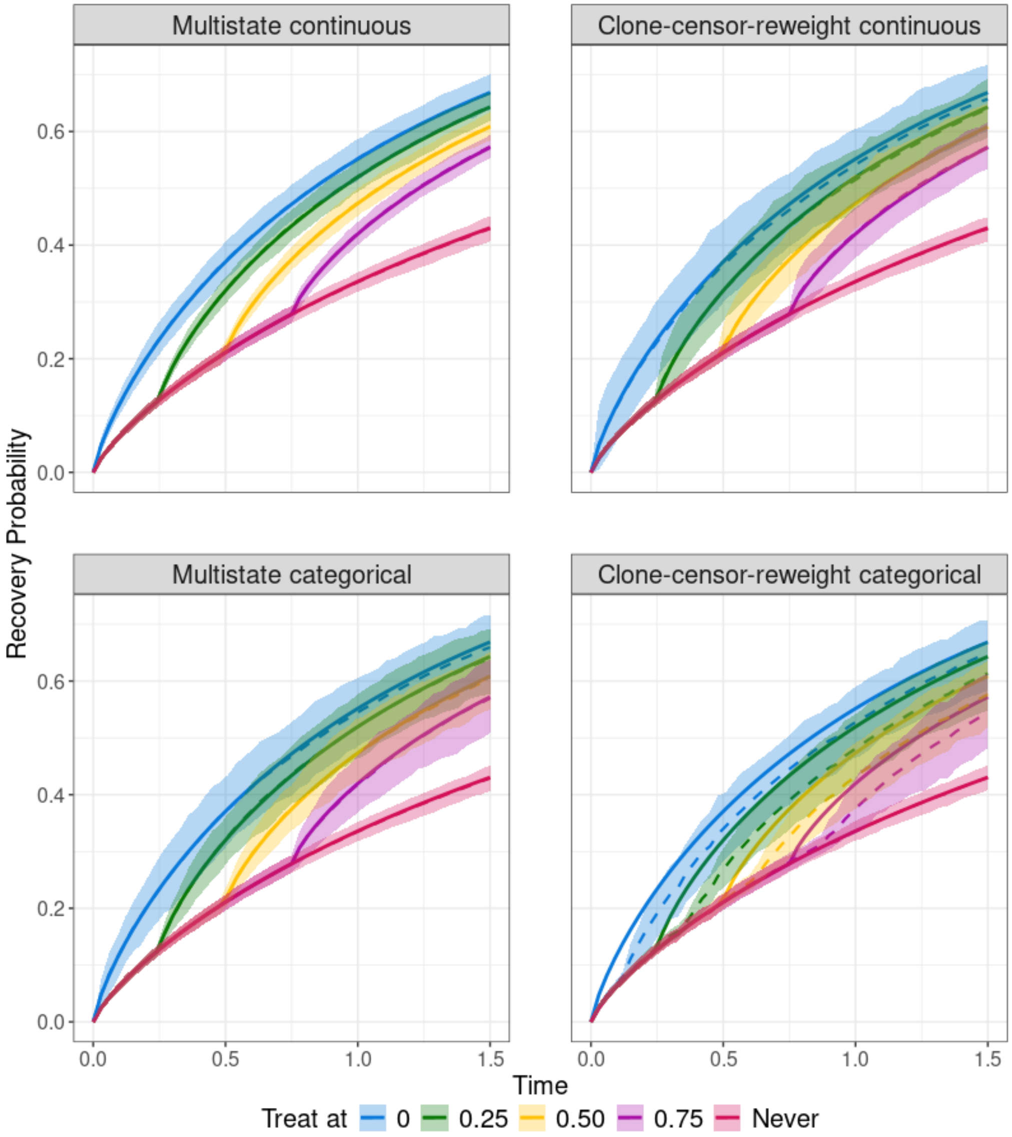

Figure 8: Visual representation of the simulation results for scenario 2. Cumulative recovery probabilities until time 1.5 are plotted, under five treatment strategies: initiate immediately, delayed at 0.25, delayed at 0.5, delayed at 0.75, and never initiate. Four estimation approaches are reported: (i) *Multistate continuous*; (ii) *Multistate categorical*; (iii) *Clone-censor-reweight continuous*; (iv) *Clone-censor-reweight categorical*. The solid lines represent the true values, the dotted line represents the mean of the estimates and the shaded area represents the $[5th - 95th]$ percentile of the estimates.

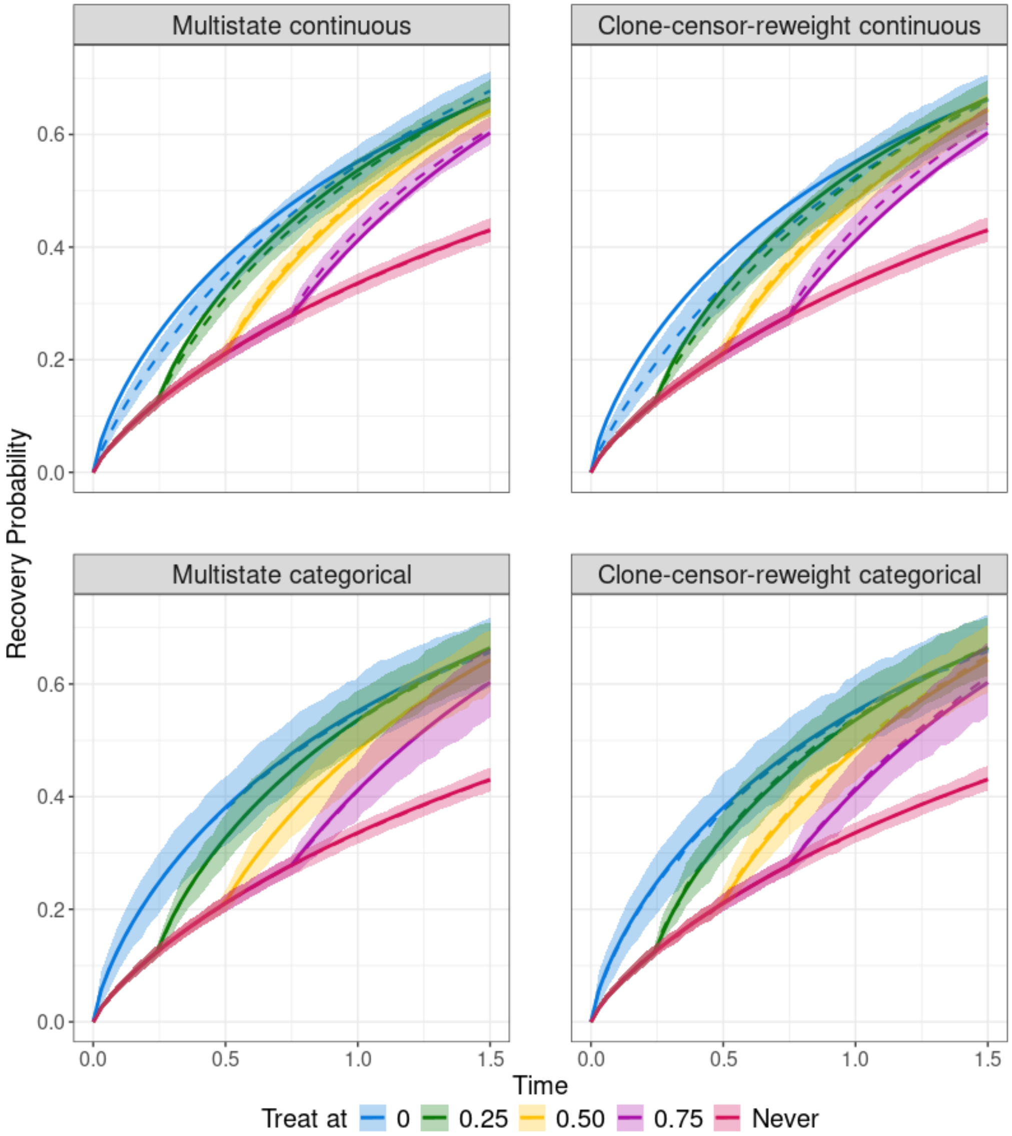

Figure 9: Visual representation of the simulation results for scenario 3. Cumulative recovery probabilities until time 1.5 are plotted, under five treatment strategies: initiate immediately, delayed at 0.25, delayed at 0.5, delayed at 0.75, and never initiate. Four estimation approaches are reported: (i) *Multistate continuous*; (ii) *Multistate categorical*; (iii) *Clone-censor-reweight continuous*; (iv) *Clone-censor-reweight categorical.*. The solid lines represent the true values, the dotted line represents the mean of the estimates and the shaded area represents the $[5th - 95th]$ percentile of the estimates.

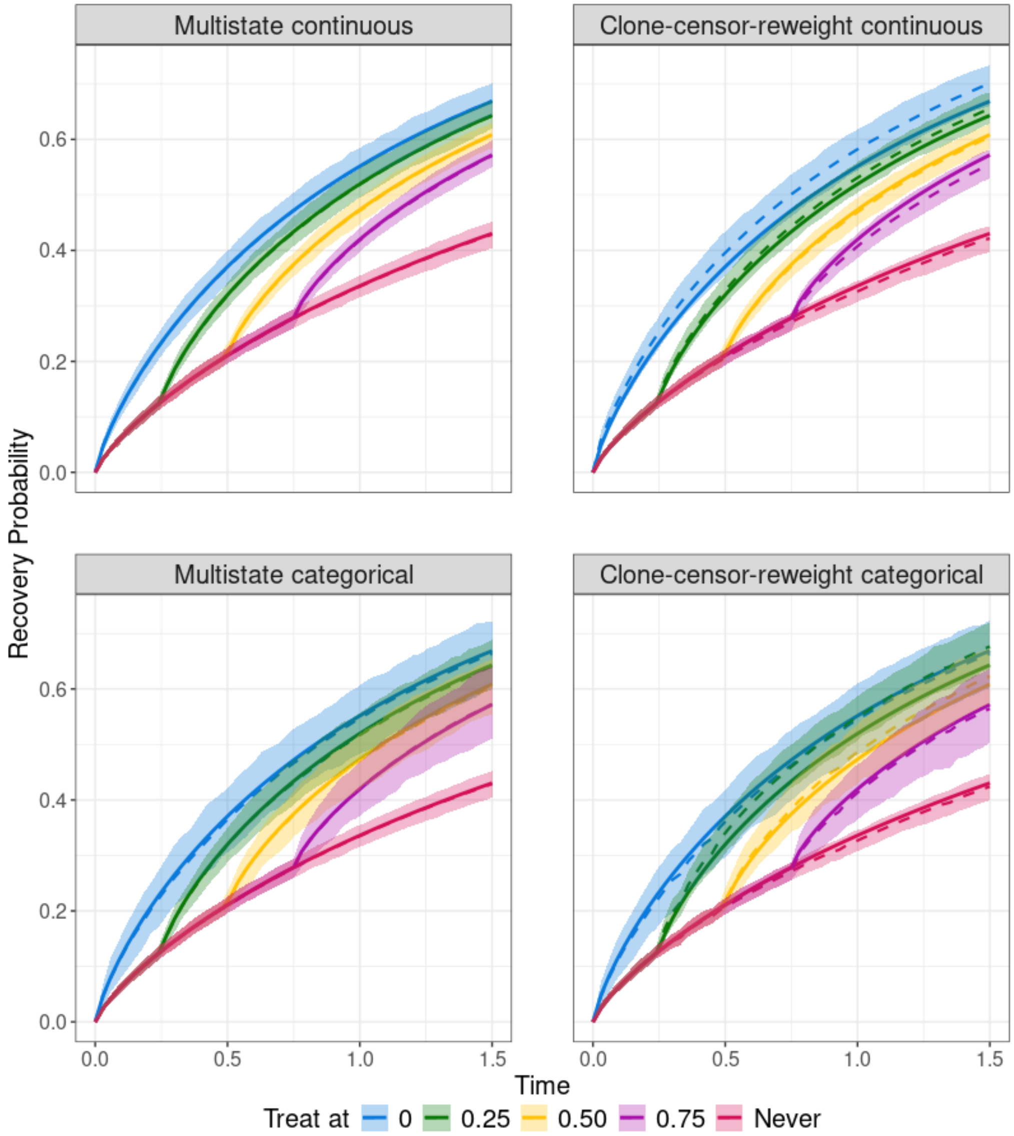

Figure 10: Visual representation of the simulation results for scenario 4. Cumulative recovery probabilities until time 1.5 are plotted, under five treatment strategies: initiate immediately, delayed at 0.25, delayed at 0.5, delayed at 0.75, and never initiate. Four estimation approaches are reported: (i) *Multistate continuous*; (ii) *Multistate categorical*; (iii) *Clone-censor-reweight continuous*; (iv) *Clone-censor-reweight categorical.* The solid lines represent the true values, the dotted line represents the mean of the estimates and the shaded area represents the $[5th - 95th]$ percentile of the estimates.

B Data application

B.1 Cumulative probabilities of first events

As a further description of our data, we provide here the cumulative probabilities over time of pregnancy without treatment and of IUI initiation.

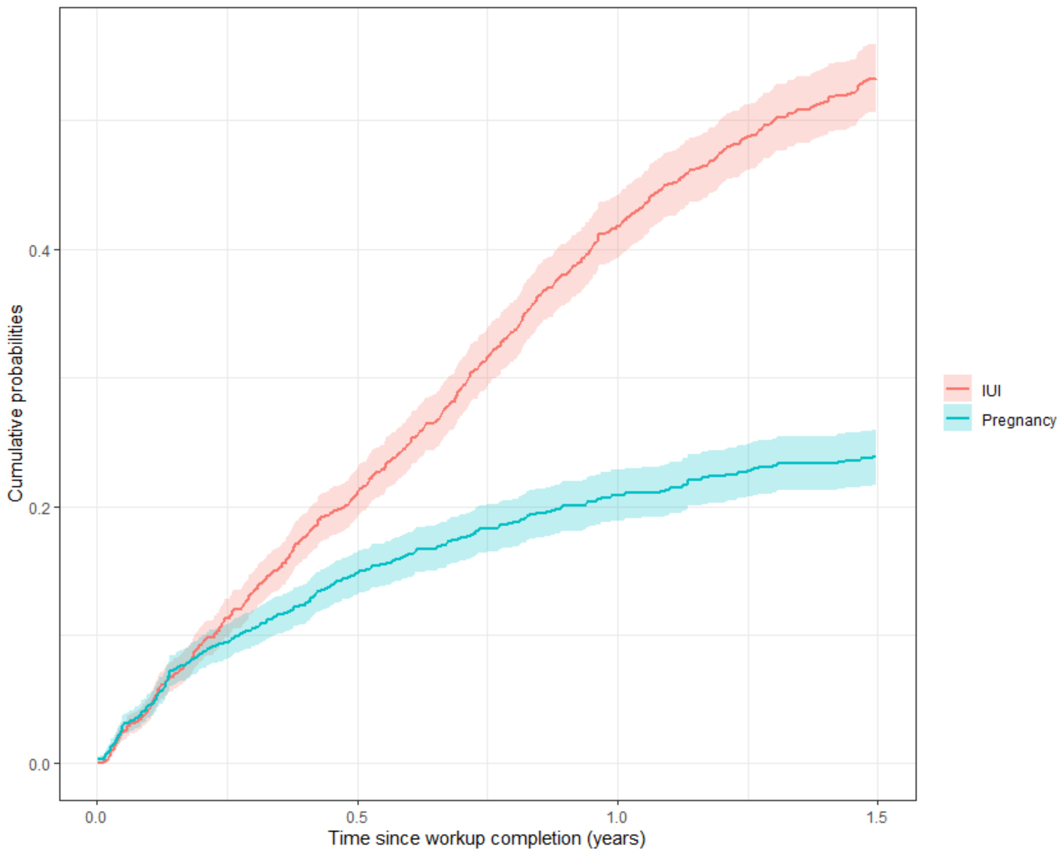

Figure 11: Cumulative probabilities over time of pregnancy without treatment and of IUI initiation.

B.2 Positivity

We empirically evaluated the validity of the (practical) positivity assumption, by checking whether certain types of couples never (or always) received the treatment around 0, 6 or 9 months into the follow up, as this would violate the assumption. Given the difficulties associated with inspecting space coverage in more than two dimensions, we conducted a pair-wise analysis of space coverage for covariates. Given the low mutual correlations among covariates, this should be enough to assess whether the positivity assumption holds.

From the visual inspection provided by Figure 12, 13 and 14, the pair-wise space seems to be covered well enough, especially if we keep in mind that we borrow information regarding the effect of the covariates on the outcome across different treatment strategies. Only few pairs of covariates have limited representatives in the data set (gynecologist referral - infertility type, gynecologist referral - tubal blockage), potentially impacting the accuracy of our model's predictions for the conditional recovery probabilities of these couples. This may affect our estimand of interest, as it is a weighted average of all the conditional recovery probabilities.

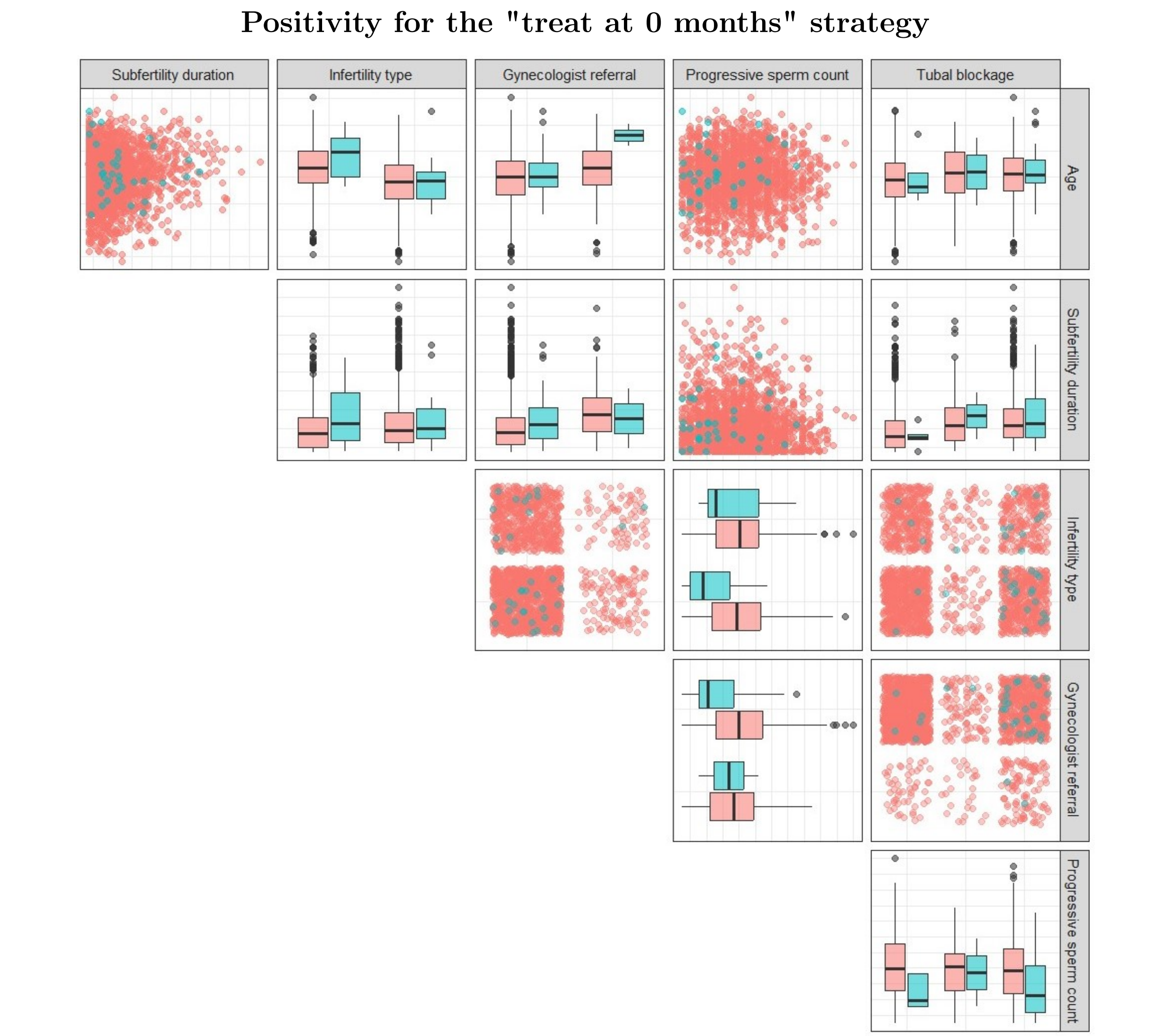

Figure 12: Pair-wise analysis of space coverage for the covariates in the multistate model, to check the validity of the positivity assumption. We use scatter plots for couples of numeric covariates, boxplots for couples of numeric-categorical covariates, jitter plots for couple of categorical covariates. Couples who followed the indicated treatment strategy are blue, while all remaining couples in the data set are red.

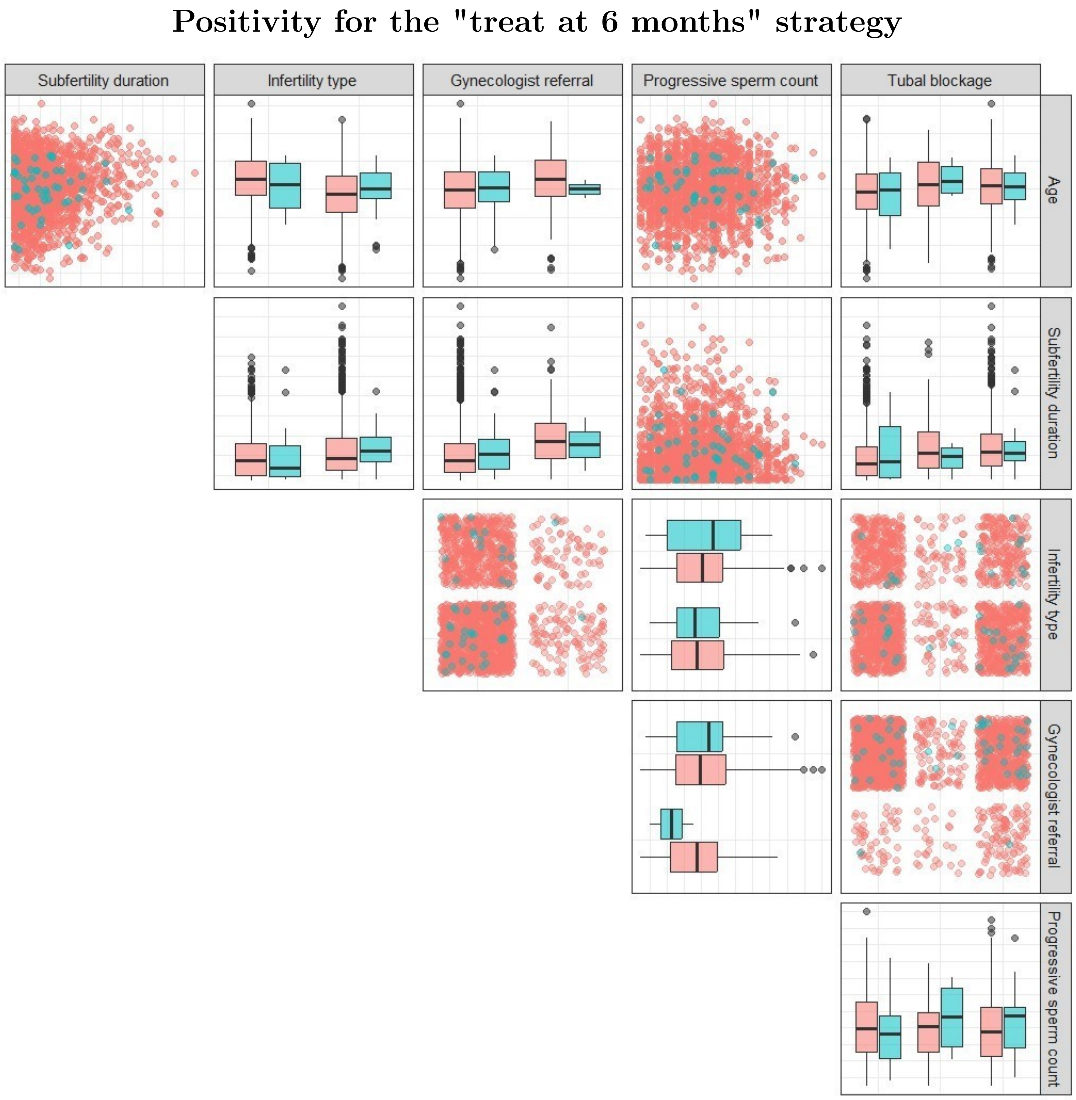

Figure 13: Pair-wise analysis of space coverage for the covariates in the multistate model, to check the validity of the positivity assumption. We use scatter plots for couples of numeric covariates, boxplots for couples of numeric-categorical covariates, jitter plots for couple of categorical covariates. Couples who followed the indicated treatment strategy are blue, while all remaining couples in the data set are red.

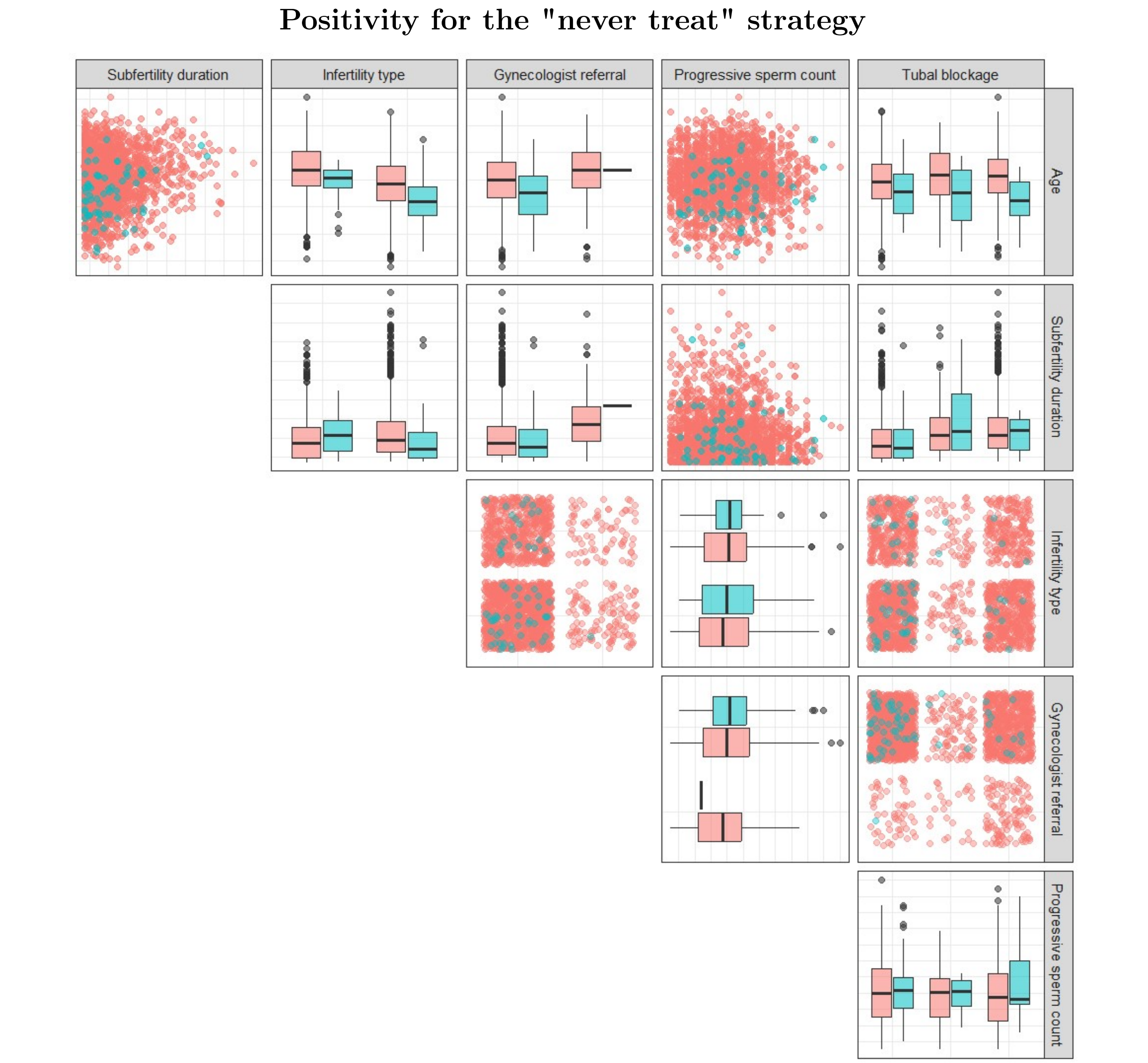

Figure 14: Pair-wise analysis of space coverage for the covariates in the multistate model, to check the validity of the positivity assumption. We use scatter plots for couples of numeric covariates, boxplots for couples of numeric-categorical covariates, jitter plots for couple of categorical covariates. Couples who followed the indicated treatment strategy are blue, while all remaining couples in the data set are red.

B.3 Follow-up completeness

We report the figures of the reverse Kaplan-Meier for transition $1 \to 3$ and $2 \to 3$. We notice that the follow-up distribution function decreases fast, with a median time of less than 1 year for both transitions. For transition $1 \to 3$ there are still 192 subjects in the at risk set at 1.5 year, leading to safely assume stable estimates of survival until time horizon. For transition $2 \to 3$ there are only 17 subjects in the at risk set at 1.5 year. This leads to more

unstable estimates of the probability of recovery under the "treat at 0" strategy towards the end (close to 1.5 years).

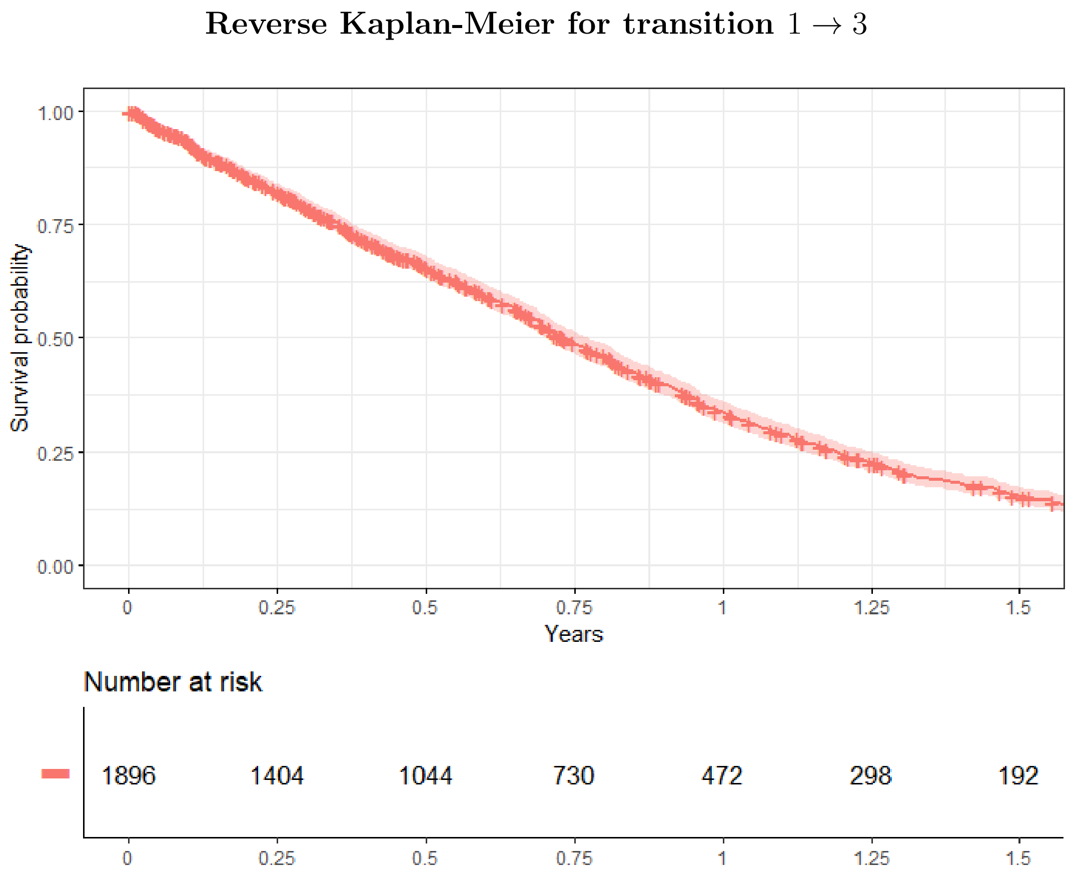

Figure 15: Follow-up distribution of transition $1 \to 3$, from baseline to time horizon (1.5 years).

Reverse Kaplan-Meier for transition $2 \to 3$

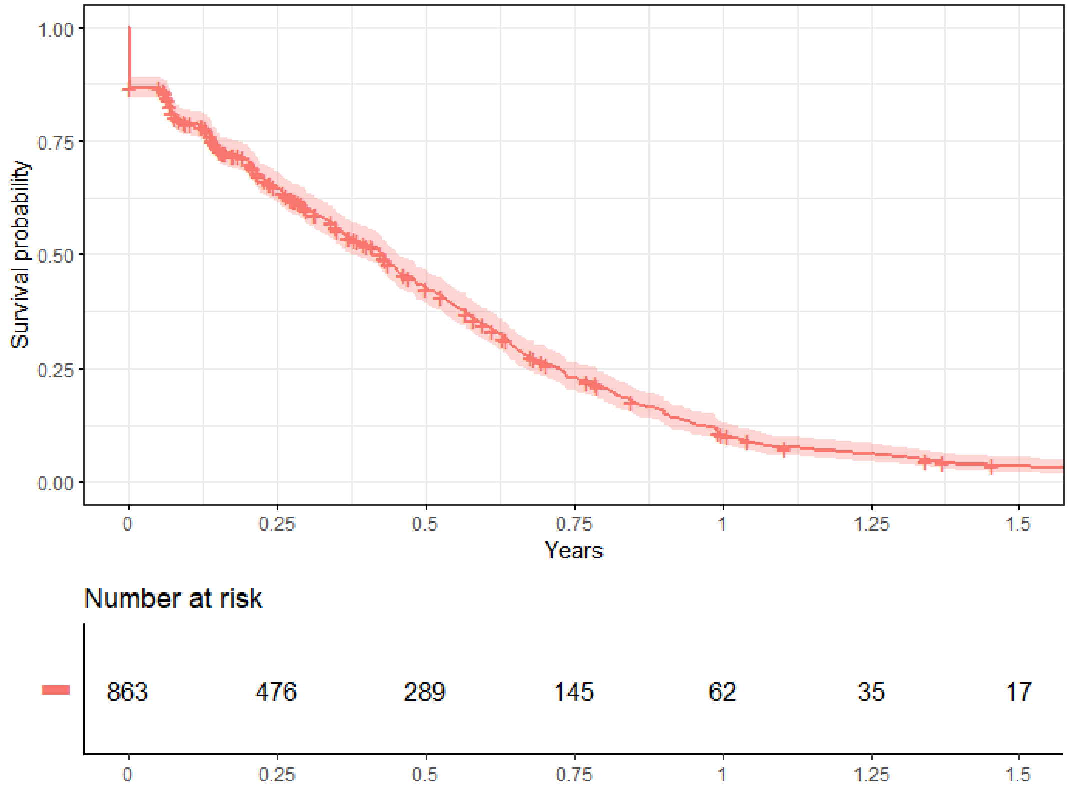

Figure 16: Follow-up distribution of transition $2 \to 3$, from treatment initiation (time 0) to 1.5 years after treatment initiation.

B.4 Proportional hazards

	Transition 1 → 3			Transition 2 → 3		
	Chi Square	df	p-value	Chi Square	df	p-value
Age	0.03	1	0.87	0.73	1	0.39
Sub-fertility duration	0.00	1	0.96	0.03	1	0.86
Infertility type	0.37	1	0.54	1.95	1	0.16
Gynecologist referral (yes/no)	0.98	1	0.32	0.23	1	0.63
Progressive sperm count	1.44	1	0.23	0.12	1	0.73
Tubal blockage "none"	0.99	1	0.32	1.73	1	0.19
Tubal blockage "1-sided"	0.01	1	0.94	0.73	1	0.39
Treatment delay				3.44	1	0.06
GLOBAL	4.19	7	0.76	9.76	8	0.28

Table 2: Chi-square statistics, degrees of freedom (df) and p-value for Schoenfeld residuals over time for the transitions 1 → 3 and 2 → 3. Tubal blockage is a 3 levels covariate with options "no blockage", "1-sided blockage" and "no test".

B.5 Linearity

We report the figures of the martingale residuals for testing the linearity between the log hazards and continuous covariates for transition $1 \to 3$ and $2 \to 3$. We do not detect any significant deviation to the linearity assumption.

Linearity of the continuous covariates for transition $1 \to 3$

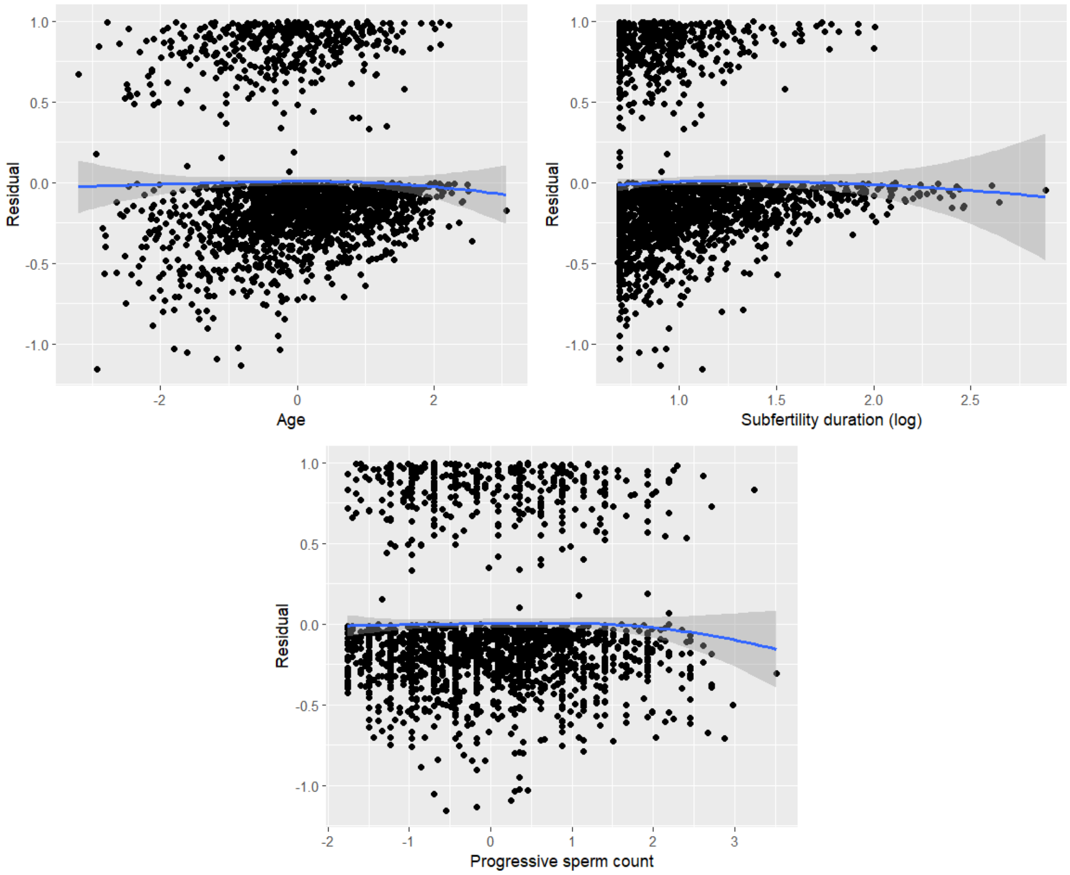

Figure 17: Martingale residuals for testing the linearity between the log hazards and continuous covariates for transition $1 \to 3$.

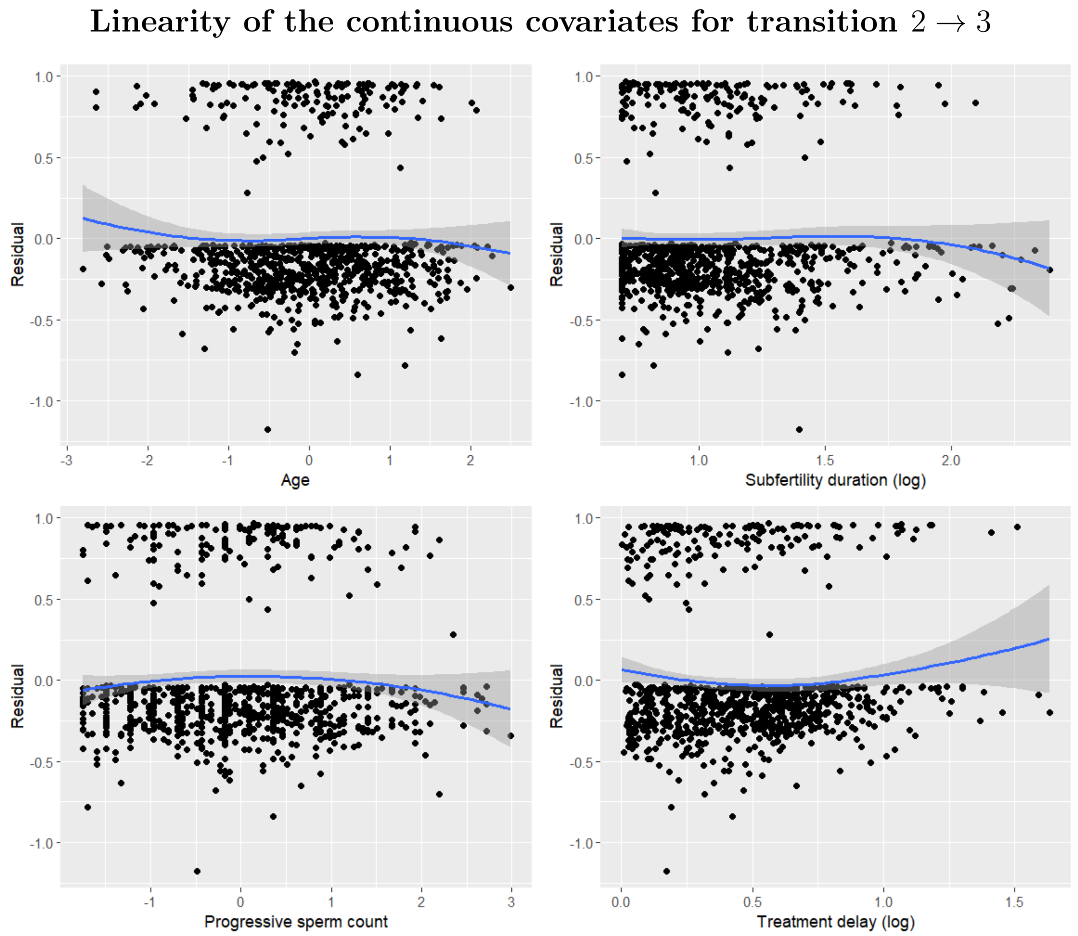

Figure 18: Martingale residuals for testing the linearity between the log hazards and continuous covariates for transition $2 \to 3$.
